# Probing lncRNA–Protein Interactions: Data Repositories, Models, and Algorithms

**DOI:** 10.3389/fgene.2019.01346

**Published:** 2020-01-31

**Authors:** Lihong Peng, Fuxing Liu, Jialiang Yang, Xiaojun Liu, Yajie Meng, Xiaojun Deng, Cheng Peng, Geng Tian, Liqian Zhou

**Affiliations:** ^1^ School of Computer Science, Hunan University of Technology, Zhuzhou, China; ^2^ Department of Sciences, Genesis (Beijing) Co. Ltd., Beijing, China; ^3^ College of Computer Science and Electronic Engineering, Hunan University, Changsha, China

**Keywords:** lncRNA–protein interaction, computational method, network-based method, machine learning-based method, data repositories

## Abstract

Identifying lncRNA–protein interactions (LPIs) is vital to understanding various key biological processes. Wet experiments found a few LPIs, but experimental methods are costly and time-consuming. Therefore, computational methods are increasingly exploited to capture LPI candidates. We introduced relevant data repositories, focused on two types of LPI prediction models: network-based methods and machine learning-based methods. Machine learning-based methods contain matrix factorization-based techniques and ensemble learning-based techniques. To detect the performance of computational methods, we compared parts of LPI prediction models on Leave-One-Out cross-validation (LOOCV) and fivefold cross-validation. The results show that SFPEL-LPI obtained the best performance of AUC. Although computational models have efficiently unraveled some LPI candidates, there are many limitations involved. We discussed future directions to further boost LPI predictive performance.

## Introduction

Long non-coding RNAs (lncRNAs) are transcripts with greater than 200 nucleotides but lack protein coding capacity (Sanchez Calle et al., 2018). lncRNAs are closely associated with various key biological processes, such as cell cycle regulation, immune response, and embryonic stem cell pluripotency (Liu et al., 2018; Agirre et al., 2019; Li et al., 2019b). More importantly, lncRNAs play an important role in understanding pathogenesis of various diseases, especially tumors (Chen et al., 2016a; Fu et al., 2017; Jiang et al., 2018; He et al., 2018a; Dallner et al., 2019). Although lncRNAs play a spectrum of regulatory roles across different cellular pathways, understanding about their regulatory mechanisms is very limited (Munschauer et al., 2018).

Recently, one broad theme is that lncRNAs can drive the assembly of RNA–protein complexes by facilitating the regulation of gene expression (Rinn and Chang, 2012; Chen and Yan, 2013; Hentze et al., 2018; Munschauer et al., 2018; Nozawa and Gilbert, 2019). lncRNAs achieve their specific functions by interacting with multiple proteins and thus regulating multiple cellular processes (Zhang et al., 2018c; Pyfrom et al., 2019). Studies reported that lncRNAs can activate post-transcriptional gene regulation, splicing, and translation by binding to proteins (Zhang et al,. 2018c; Li et al., 2019a) Therefore, identifying possible lncRNA–protein interactions (LPIs) is essential for unraveling lncRNA-related activities (Qian et al., 2018; Zhang et al., 2018c; Zhao et al., 2018c). Wet experiments validated parts of LPIs, but experimental methods remain costly and time-consuming. Therefore, different computational models are explored to infer potential LPIs (Pan et al., 2016; Cheng et al., 2018; Zhang et al., 2018c; Zhao et al., 2018c). There exist numerous unexplored lncRNAs and proteins in public databases, which makes it possible to efficiently identify their underlying associations.

In this study, we introduced relevant repositories, summarized computational models and algorithms for LPI prediction, discussed their advantages and weaknesses by comparison, and presented further directions for boosting LPI prediction performance. We focused on two categories of computational models: network-based methods and machine learning-based methods. The machine learning-based methods contain matrix factorization-based methods and ensemble learning-based methods.

## Relevant Repositories

There are abundant repositories related to LPI prediction. These repositories provide diverse information for efficiently uncovering potential LPIs.

### Noncode

The NONCODE database (Zhao et al., 2015) (http://www.noncode.org/) is an interactive database aiming to collect the most complete annotation for ncRNAs, especially lncRNAs. The latest NONCODE database (current version v5.0) contains lncRNA information from 17 species including human, mouse, cow, rat, chimp, gorilla, orangutan, rhesus, opossum, platypus, chicken, zebrafish, fruit fly, *Caenorhabditis elegans*, yeast, Arabidopsis, and pig. There are 548,640 lncRNAs in the latest version. There are 172,216 and 131,697 lncRNAs from human and mouse, respectively. More importantly, NONCODE has introduced some important features including conservation annotation, lncRNA–disease associations, and an interface to select credible datasets.

### NPInter

The NPInter database (Hao et al., 2016) (http://www.bioinfo.org.cn/NPInter/contact.htm) provides abundant association data that are experimentally verified. For example, the database contains information on interactions between noncoding RNAs (ncRNAs) and biomolecules including proteins, mRNAs, miRNAs, and genomic DNAs. The database contains 491,416 interactions in 188 tissues/cell lines from 68 types of experimental technology.

### RAID

The RAID database (Yi et al., 2016) (http://www.rna-society.org/raid/) includes more than 40,668 lncRNA-associated RNA–protein interactions and more than 34,790 lncRNA-associated RNA–RNA interactions.

### starBase

The starBase database (Li et al., 2013) (http://starbase.sysu.edu.cn/) contains more than 1,100,000 miRNA–ncRNA (CLIP) interactions, 117,000 RNA-binding protein (RBP)–ncRNA interactions, and 32,000 miRNA–ncRNA interactions. In addition, it provides more than 10,800 RNA-seq data and 10,500 miRNA-seq data from 32 cancer types and 3,236,000 mutations from 366 disease types.

### VirBase

The ViRBase database (Li et al., 2014) (http://www.rna-society.org/virbase) integrates experimental and predictive association information from manual literature curation and other resources based on one common framework from 119 species, especially ncRNA-associated virus–virus, host–host, host–virus, and virus–host interactions.

### POSTAR2

The POSTAR2 database (Zhu et al., 2018) (http://lulab.life.tsinghua.edu.cn/postar2/index.php) provides various post-transcriptional regulation data based on CLIP-seq, Ribo-seq, RNA-seq, and other high-throughput sequencing information from six species: yeast, Arabidopsis, fly, worm, mouse, and human. It hosts about 40 million RBP binding sites validated by CLIP-seq experiments. It provides three modules: the “RBP” module, “RNA” module, and “Translatome” module. The “RBP” module contains RBP binding sites and their annotations and functions. The “RNA” module is composed of a few sub-modules, including “disease,” “variation,” “crosstalk,” and “binding sites,” and is applied to annotate the RBP binding sites.

### ChIPBase

The ChIPBase database (Zhou et al., 2016) (http://rna.sysu.edu.cn/chipbase/) is used to identify transcription factor binding sites and motifs, and decode transcriptional regulatory networks of miRNA, lncRNAs, and other ncRNAs from ChIP-seq data. It provides about 10,200 curated peak datasets from 10 species: human, mouse, fruit fly, worm, *Arabidopsis thaliana*, yeast, rat, zebrafish, *Xenopus tropicalis*, and chicken.

### LNCipedia

The LNCipedia database (Volders et al., 2018) (https://lncipedia.org) is a comprehensive database. Its central work is to merge redundant transcripts from different data sources and group the transcripts into genes, thus producing a highly consistent database. The latest update of lncRNA (LNCipedia 5) contains information about annotation and sequence for 1,555 human lncRNAs from 2,482 lncRNA publications. This information originates from Ensembl (Cunningham et al., 2018), RefSeq (Rajput et al., 2018), and FANTOM CAT (Hon et al., 2017).

### lncRNA2target

The lncRNA2Target database (Cheng et al., 2018) (http://123.59.132.21/lncrna2target) contains a comprehensive repository of lncRNA target genes to provide information about target genes regulated by lncRNAs. The latest version provides a special web interface in which users can search the targets for a particular lncRNA or the lncRNAs for a particular gene.

### lncRNAdb

The lncRNAdb database (Quek et al., 2014) (http://lncrnadb.org) is a comprehensive database in compliance with the International Nucleotide Sequence Database Collaboration. It provides 287 eukaryotic lncRNAs and an interface enabling users to access sequence data, expression information, and the literature. The latest update of lncRNAdb integrated nucleotide sequence information, Illumina Body Atlas expression profiles, and a BLAST search tool.

### lncRNASNP2

The lncRNASNP2 database (Miao et al., 2017) (http://bioinfo.life.hust.edu.cn/lncRNASNP2) provides 7,260,238 single nucleotide polymorphisms (SNPs) on 141,353 human lncRNA transcripts, and 3,921,448 SNPs on 117,405 mouse lncRNA transcripts. More importantly, it contains abundant information about mutations in lncRNAs and their impacts on lncRNA structure and function. It also provides online tools for analyzing new variants in lncRNA.

### LbcRNAwiki

The lncRNAWiki database (Ma et al., 2014) (http://lncrna.big.ac.cn) integrated various human lncRNAs from different resources. It makes existing lncRNAs able to be updated, edited, and curated by diverse users. More importantly, any user can add newly uncovered lncRNAs.

### Lnc2Cancer

The Lnc2Cancer database (Gao et al., 2018) (http://www.bio-bigdata.net/lnc2cancer) provides lncRNA–cancer associations supported by experiments. It contains 4,989 associations between 165 human cancer subtypes and 1,614 human lncRNAs, 366 experimentally validated circulating-related lncRNA-cancer associations, 593 drug-resistance-related lncRNA-cancer associations, and 1,928 prognosis-related lncRNA–cancer associations, and abundant lncRNA regulatory mechanisms in cancers including 211, 1139, 225, and 319 lncRNAs regulated by variant, miRNA, transcription factor, and methylation, respectively.

### LncRNAdisease

The lncRNADisease database (Bao et al., 2018) (http://www.rnanut.net/lncrnadisease/) integrated experimentally validated circular RNA–disease associations, and regulatory mechanisms among mRNA, miRNA, and ncRNA. Particularly, it contains more than 200, 000 lncRNA–disease associations. In addition, it gives confidence scores for all ncRNA–disease associations and maps each disease to disease ontology and medical subject headings.

### MNDR

The MNDR database (Cui et al., 2017) (http://www.rna-society.org/mndr/) integrates more than 260,000 ncRNA–disease associations. These associations are supported by 10 experiments and 4 predictive algorithms. The experimental repositories include Lnc2Cancer (Gao et al., 2018), dbDEMC (Yang et al,. 2016), LncRNADisease (Bao et al., 2018), MNDR (Wang et al., 2013), HMDD (Huang et al., 2018b), NSDNA (Wang et al., 2016a), LincSNP (Ning et al., 2016), miRCancer (Xie et al., 2013), PhenomiR (Ruepp et al., 2012), and miR2Disease (Jiang et al., 2008). The four prediction algorithms are LDAP (Lan et al., 2016), miRDP (Mørk et al., 2013) LncDisease (Wang et al., 2016b), and PBMDA (You et al., 2017). It provides 8,824 experimental lncRNA–disease, 70,381 experimental miRNA–disease, 118 experimental piRNA–disease, and 67 experimental snoRNA–disease associations across 6 mammalsix (*Homo sapiens*, *Macaca mulatta*, *Mus musculus*, *Pan troglodyte*, *Rattus norvegicus*, and *Sus scrofa*). In addition, it provides 153,508 predicted lncRNA–disease associations and 28,144 predicted miRNA–disease associations for *H. sapiens*. MNDR contains 19,575, 110, 4,150, and 23 non-redundant lncRNA–disease, piRNA–disease, miRNA–disease, and snoRNA–disease interactions, respectively, associated with 1,416 disease.

### UniProt

The UniProt database (Consortium et al., 2018) (http://www.uniprot.org/) is an important database providing protein sequences and annotations. It provides 80 million sequences and is a useful tool. Users can calculate a new proteome identifier to find a particular assembly for a species or subspecies. It also provides an effective measurement for computing an annotation score for all entries.

## Methods

Most computational methods contain two procedures: data extraction and model selection. In the first part, computational methods usually extract LPIs related to human lncRNA, lncRNA sequences, and protein sequences from NPInter (Hao et al., 2016), NONCODE (Zhao et al., 2015), and UniProt (Consortium et al., 2018), respectively. Computational methods filter LPIs by removing lncRNAs/proteins only interacting with one protein/lncRNA. In the second procedure, computational methods design various models to uncover potential LPIs. These models can be roughly classified into two categories: network-based methods and machine learning-based methods.

### Data Representation

Computational methods utilize an lncRNA set *l* *=* {*l*_1_ *, l*_2_ *, l*_3_ …. *l_n_*}, a protein set, *P* = {*p*_1_, *p*_2_, *p*_3_ …. *p_m_*}, and an LPI matrix *Y_n×m_*, where *y_ij_* = 1 if there is an association between an lncRNA *l_i_* and a protein *p_j_*; otherwise, *y_ij_* = 0.

### Network-Based Methods

Network-based methods obtain better performance by effectively integrating related biological information and network propagation algorithms into a unified framework.

#### LPIHN

Li et al. (2015) developed an LPI prediction method combing a heterogeneous network model and random walk with restart, LPIHN. LPIHN can be broken down into four steps:

Step 1 Extracting known ncRNA–protein associations from the Npinter 2.0 database (Hao et al., 2016) and filtering the ncRNAs and their associated proteins based on organism and type of ncRNAs. LPIHN then selects lncRNAs from filtered ncRNAs based on the human lncRNA dataset provided by the NONCODE database (Zhao et al., 2015).

Step 2 Obtaining lncRNA expression profiles from the NONOCODE 4.0 database (Zhao et al., 2015). Given the expression profiles of two lncRNAs *E*_1_ and *E*_2_, LPIHN calculates lncRNA expression similarity based on the Pearson correlation coefficient:

(1) $$SL(i,j)=\left|\frac{\mathrm{cov}(E_{1},E_{2})}{\sigma_{e_{1}}\sigma_{e_{2}}}\right|$$

where *cov*(*E*_1_, *E*_2_) is the covariance of *E*_1_ and *E*_2_, and $\sigma_{e_{1}}$ and $\sigma_{e_{2}}$ are the standard deviations of *E*_1_ and *E*_2_, respectively.

Step 3 Extracting protein–protein interactions (PPIs) from STRING 9.1 (Szklarczyk et al., 2016) and obtaining 804 PPIs and the corresponding score matrix *SP*. *SP* is normalized as follows:

(2) $$SP_{ij}^{*}=\frac{SP_{ij}}{\sqrt{M\left(i,i\right)M\left(j,j\right)}}$$

where *M* is a diagonal matrix, and *M*(*i*, *i*) is the sum of row *i* in *SP*.

Step 4 Propagating the random walk to score for unknown lncRNA–protein pairs based on the following iterative equation:

(3) $$Y_{t+1}=(1-\delta )W^{T}Y_{t}+\delta \;Y_{0}$$

**Figure 1:**
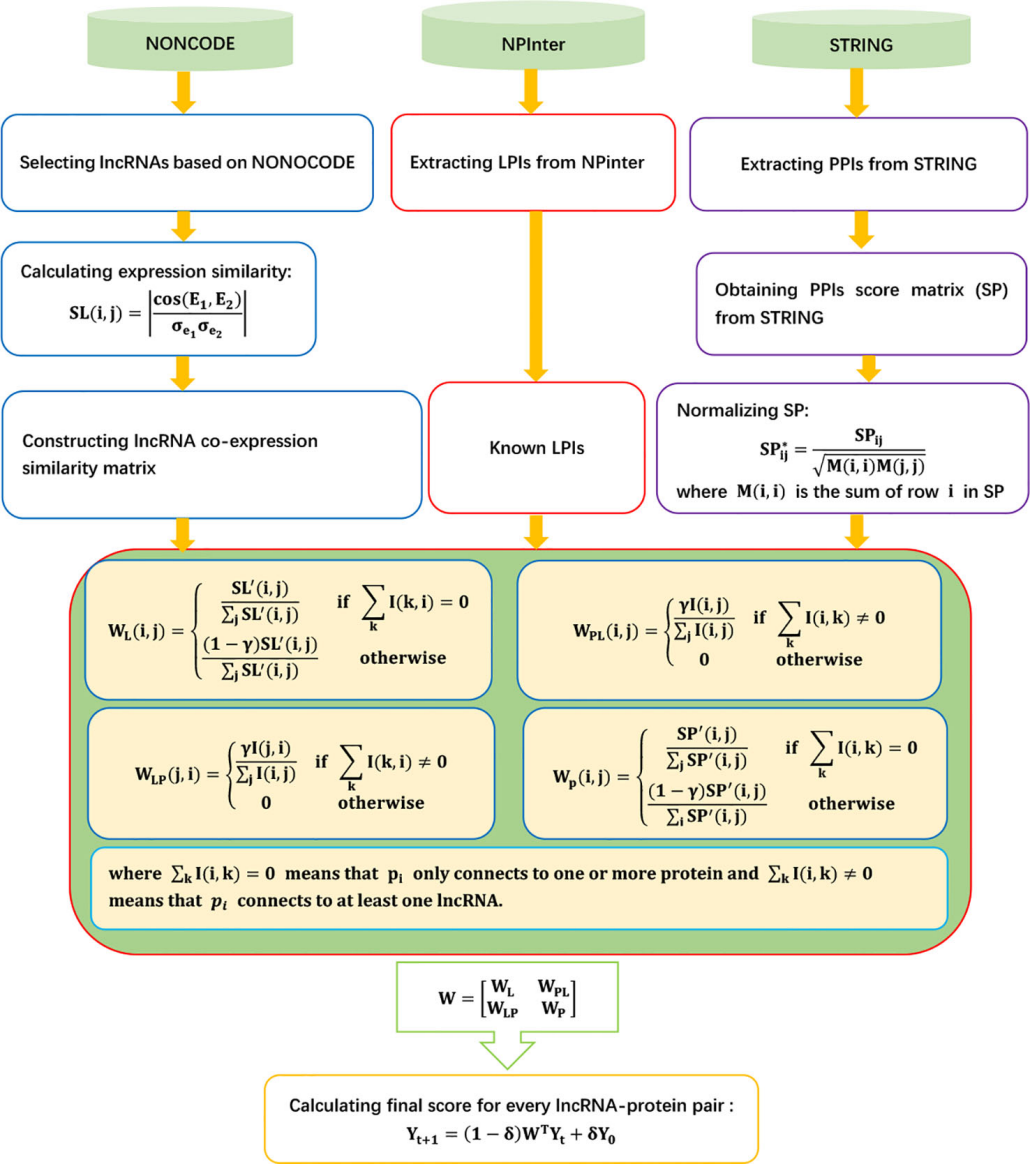
Flowchart of LPI prediction method based on heterogeneous network model and random walk with restart.

The details are shown as **Figure 1**.

#### LPLNP

Zhang et al. (2018b) proposed a linear neighborhood propagation-based method, LPLNP, to probe potential LPIs. LPLNP found novel LPIs through the following steps.

Step 1 Extracting 4,158 LPIs between 27 proteins and 990 lncRNAs from NPInter (Hao et al., 2016) and NONCODE (Zhao et al., 2015) by filtering unreliable lncRNA sequences and removing lncRNAs/proteins only interacting with one protein/lncRNA.

Step 2 Obtaining three types of features for lncRNAs (interaction profile, expression profile, and sequence composition) and two types of features for proteins [interaction profile and CTD (composition, transition, and destruction)].

Step 3 Computing linear neighborhood similarity and regularized linear neighborhood similarity between lncRNA/proteins by Eqs. (4) and (5), respectively:

(4) $$\begin{array}{l} \epsilon_{i}=\|X_{i}-\sum_{i_{j}:X_{i_{j}}\in N(X_{i})}w_{ii_{j}}X_{i_{j}}{\|}^{2}\\ s.t.\sum_{i_{j}:X_{i_{j}}\in N(X_{i})}w_{ii_{j}}=1,w_{ii_{j}}\geq 0 \end{array}$$

where *X_i_* denoted the feature vector of the *i*th lncRNA, and *N*(*X_i_*) is *K* nearest neighbors of *X_i_*.

(5) $$\begin{array}{l} \epsilon_{i}=w_{i}^{T}(G^{i}+\lambda I)w_{i}\\ s.t.\sum_{i_{j}:X_{i_{j}}\in N(X_{i})}w_{ii_{j}}=1,w_{ii_{j}}\geq 0 \end{array}$$

where $G_{i_{j}i_{k}}={\left(X_{i}-X_{i_{j}}\right)}^{T}(X_{i}-X_{i_{j}})$.

Step 4 Computing the interaction probabilities for unobserved lncRNA–protein pairs:

(6) $$Y=(1-\alpha ){\left(I-\alpha W\right)}^{-1}Y^{0}$$

**Figure 2:**
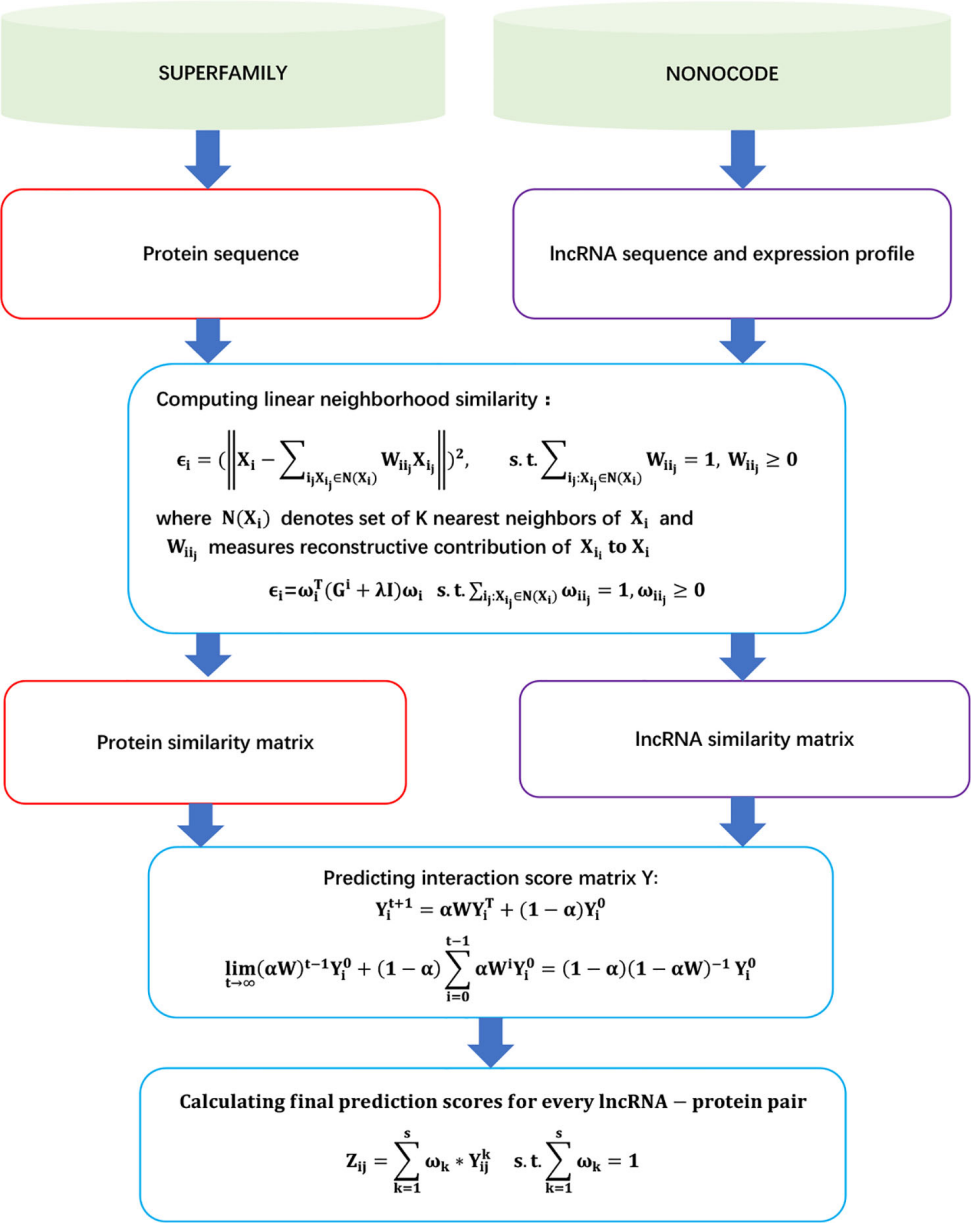
Flowchart of linear neighborhood propagation-based LPI prediction method.

The details are shown in **Figure 2**.

#### LPI-BNPRA

Zhao et al. (2018a) developed a novel LPI prediction model based on a bipartite network projection recommended technique, LPI-BNPRA. LPI-BNPRA can be broken down into five steps.

Step 1 Extracting 4,158 high-confidence LPIs between 990 lncRNAs and 27 proteins from NPInter (Hao et al., 2016) and NONCODE (Zhao et al., 2015) by filtering unreliable lncRNA sequences and removing lncRNAs/proteins only associated with one protein/lncRNA.

Step 2 Calculating lncRNA–lncRNA similarity based on the Smith–Waterman technique:

(7) $$LSM\left(l_{i},l_{j}\right)=\frac{sw\left(l_{i},l_{j}\right)}{\max\;(sw\left(l_{i},l_{i}\right),sw\left(l_{j},l_{j}\right))}$$

where *sw*(*l_i_, l_j_*) denotes the Smith–Waterman score between two lncRNAs *l_i_* and *l_j_*.

Step 3 Calculating the protein–protein similarity matrix based on the Smith–Waterman technique:

(8) $$PSM(p_{i},p_{j})=\frac{sw(p_{i},p_{j})}{\max(sw(p_{i},p_{i}),sw(p_{j},p_{j}))}$$

where *sw*(*p_i_, p_j_*) denotes the Smith–Waterman score between two proteins *p_i_* and *p_j_*.

Step 4 For a given lncRNA *l_j_*, computing its bias ratings of lncRNAs for a protein *p_i_* with the agglomerative hierarchical clustering and associated measurement of minimum variance method:

(9) $$r(p_{i},l_{j})=\frac{n_{cr}}{T(p_{i})}$$

where *n_cr_* is the number of lncRNAs in the cluster *cr* including *l_j_*, and *Tp_i_* is the number of all lncRNAs interacting with *p_i_*.

Step 5 Finding LPI candidates based on the recommended bipartite network projection technique and bias ratings of every lncRNA for proteins:

(10) $$R_{fin}(l_{j})=\sum_{i=1}^{n}R_{fin}(p_{i},l_{j})$$

where

(11) $$R_{fin}(p_{i},l_{j})=\frac{r(p_{i},l_{j})}{\sum_{k=1}^{n}r(p_{k},l_{j})}\times R(p_{i})$$

(12) $$R(p_{i})=\sum_{j=1}^{m}R(p_{i},l_{j})$$

(13) $$R(p_{i},l_{j})=\frac{r_{\mathrm{ini}}(p_{i},l_{j})}{\sum_{k=1}^{n}r_{\mathrm{ini}}(p_{k},l_{j})}\times R_{ini}(l_{j})$$

(14) $$R_{ini}(l_{j})=r_{ini}(p_{i},l_{j})$$

(15) $$r_{ini}(p_{i},l_{j})=\frac{r(p_{i},l_{j})}{r_{ave}(p_{i},l_{j})}$$

(16) $$r(p_{i},l_{j})=\frac{n_{cr}}{T(p_{i})}$$

(17) $$r_{ave}(p_{i},l_{j})=\frac{\sum_{j=1}^{m}r(p_{i},l_{j})}{T(p_{i})}$$

**Figure 3:**
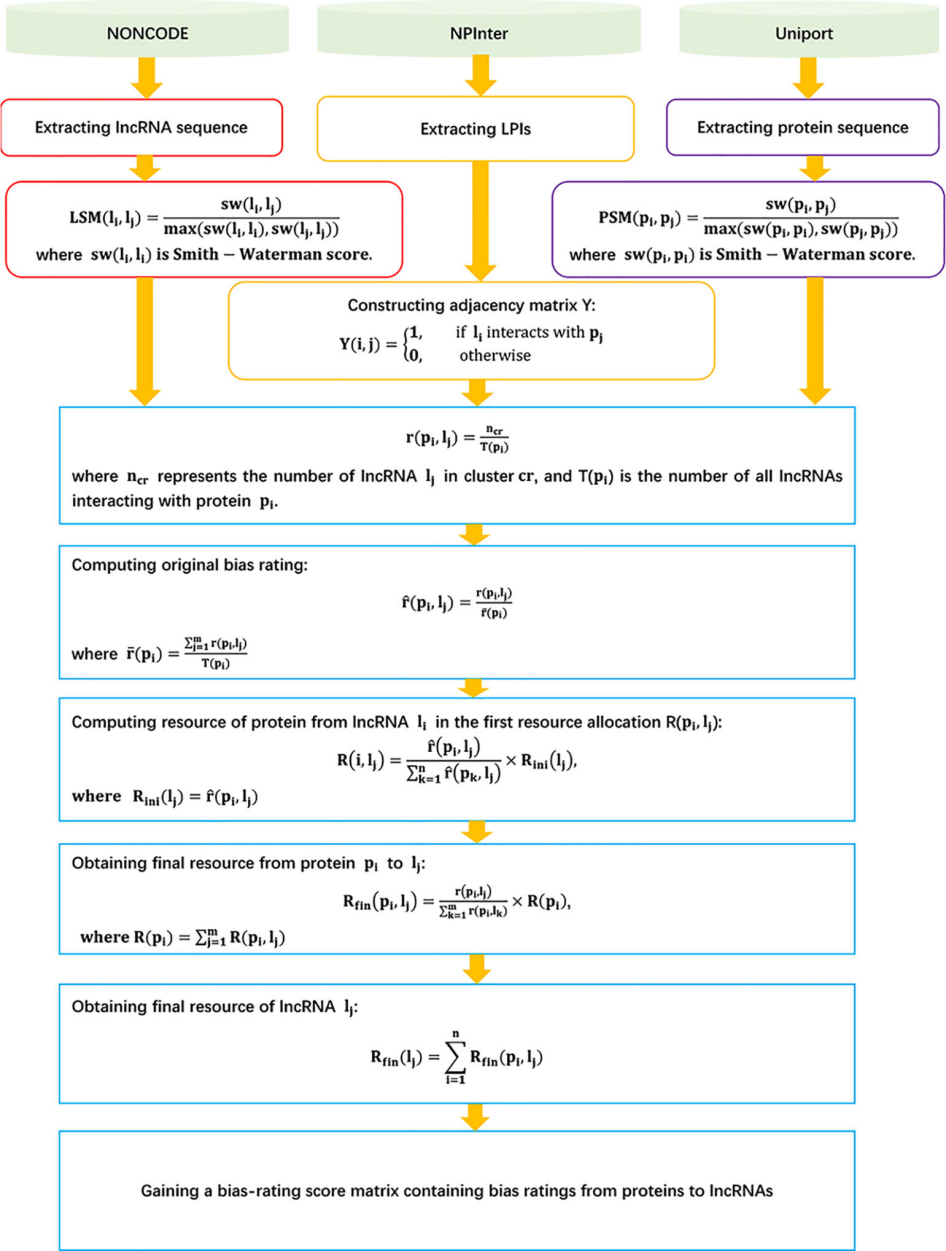
Flowchart of LPI prediction model based on the recommended bipartite network projection technique.

The details are shown in **Figure 3**.

#### LPISNFHS

Zheng et al. (2017) presented a new LPI identification method, LPISNFH. LPISNFHS fused multiple protein–protein similarity networks, the similarity network fusion (SNF) technique, HeteSim algorithm, and known LPI network into a unified framework. LPISNFH can be broken down into three steps.

Step 1 Obtaining 4,467 LPIs between 1,050 unique lncRNAs and 84 unique proteins from NPInter (Hao et al., 2016) and NONCODE (Zhao et al., 2015) by manually filtering LPIs not involving lncRNAs and removing the lncRNAs only associated with one protein.

Step 2 Constructing a protein–protein similarity network. LPISNFHS fused the sequence similarity, functional annotation semantic similarity (Go), domain similarity, and STRING similarity into a unified protein–protein similarity network based on the SNF technique.

Step 3 Inferring novel LPIs by combining the HeteSim algorithm and heterogeneous LPI network.

#### LPI-IBNRA

Xie et al. (2019) developed a LPI prediction model, LPI-IBNRA. LPI-IBNRA integrated lncRNA–protein interactions, protein–protein interactions, and similarity matrix for proteins and lncRNAs, and improved bipartite network recommender algorithm. LPI-IBNRA can be broken down into seven steps.

Step 1 Obtaining 4,796 LPIs between 1,105 lncRNAs and 26 proteins from NPInter (Hao et al., 2016) and NONCODE (Zhao et al., 2015) after filtering lncRNAs and proteins that have only one association.

Step 2 Computing lncRNA similarity matrix *sim^L^* based on lncRNA expression similarity and Gaussian interaction profile (GIP) kernel similarity, and protein similarity matrix *sim^P^* based on protein interaction similarity and GIP kernel similarity.

Step 3 Computing the score between protein *p_i_* and lncRNA *l_j_* based on protein similarity and lncRNA similarity by Eqs. (18) and (19), respectively.

(18) $$S^{P}\left(p_{i},l_{j}\right)=\{\begin{array}{c} \frac{\sum_{k=1}^{np}sim^{P}\left(p_{i},p_{k}\right)I\left(p_{k},l_{j}\right)}{\sum_{k=1}^{np}sim^{P}\left(p_{i},p_{k}\right)}\;\mathrm{if}\;I\left(p_{i},l_{j}\right)=1\\ 0\;\mathrm{otherwise} \end{array}$$

(19) $$S^{L}\left(p_{i},l_{j}\right)=\{\begin{array}{c} \frac{\sum_{k=1}^{nl}I\left(p_{i},l_{k}\right)sim^{L}\left(l_{k},l_{j}\right)}{\sum_{k=1}^{np}sim^{L}\left(l_{k},l_{j}\right)}\;\mathrm{if}\;I\left(p_{i},l_{j}\right)=1\\ 0\;\mathrm{otherwise} \end{array}$$

Step 4 Obtaining the initialized association score matrix as follows:

(20) $$S_{\mathrm{ini}}=\gamma S^{P}+\left(1-\gamma \right)S^{L}$$

Step 5 Computing the first-round scores of the lncRNA *l_k_* over all proteins:

(21) $$s_{1}\left(l_{k}\right)=\sum_{j=1}^{np}\frac{S_{\mathrm{ini}}\left(p_{j},l_{k}\right)s_{0}\left(p_{j}\right)}{d\left(p_{j}\right)}$$

Step 6 Computing the second-round scores of the protein *p_i_* over all lncRNAs:

(22) $$s_{2}\left(p_{i}\right)=\sum_{k=1}^{nl}\frac{S_{ini}\left(p_{i},l_{k}\right)}{d\left(l_{k}\right)}\sum_{k=1}^{np}\frac{S_{ini}\left(p_{j},l_{k}\right)s_{0}\left(p_{j}\right)}{d\left(p_{j}\right)}$$

Step 7 Computing the final association score matrix:

(23) $$S_{fin}^{\prime }=W^{\prime }S_{ini}$$

where *W′* = *W* + *αW* ^2^ and *α* ∈ (−1,0).

**Figure 4:**
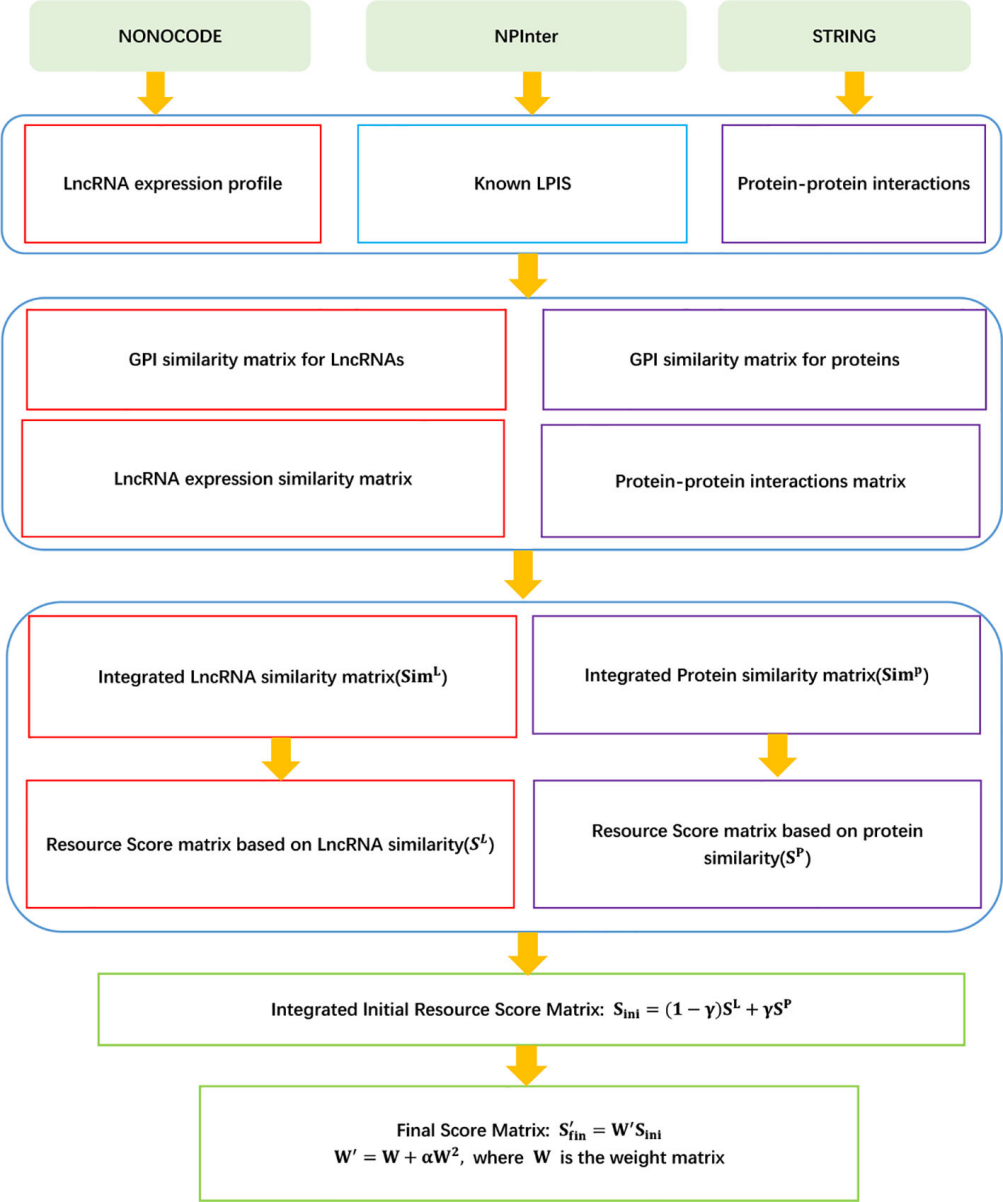
Flowchart of LPI prediction method based on improved bipartite network recommender algorithm.

The details are shown in **Figure 4**.

#### LPBNI

Ge et al. (2016) proposed an lncRNA–protein bipartite network inference method, LPBNI, to find potential LPIs. LPBNI can be broken down into five steps.

Step 1 Extracting data. LPBNI first downloads 7,576 ncRNA–protein associations from NPInter 2.0 (Hao et al., 2016) with the restricted type of “NONCODE” and organism “*Homo sapiens*.” LPBNI then selects 2,380 lncRNAs based on a human lncRNA dataset provided by the NONCODE database (Zhao et al., 2015). Finally, LPBNI extracts 4,870 LPIs between 2,380 lncRNAs and 106 proteins.

Step 2 Utilizing the LPI network to construct a bipartite graph *G* (*L*, *P*, *Y*).

Step 3 Propagating known biological information in *G*. For a lncRNA *l_j_*, *S_L_* (*l_j_*) denotes the score on *l_j_* after the first step of propagation:

(24) $$S_{L}(l_{j})=\sum_{i=1}^{m}\frac{a_{ij}S_{0}(i)}{d(p_{i})},j\in \{1,2,3\ldots .n\}$$

where *S*_0_ (*i*) = *s_ij_*, *i* ∈ {*i*, 2,…, *m*} denotes the original information of *P* for a given lncRNA *l_j_*. *s_ij__=_* 1 if *p_i_* associates with *l_j_*; otherwise, *s_ij_* = 0. $d\left(p_{i}\right)=\sum_{j=1}^{n}a_{\mathrm{ij}}$ denotes the number of lncRNAs associated with *p_i_*.

Step 4 Propagating all information in *L* back to *P*. *S_F_*(*p_i_*) represents the final information on protein *p_i_* to denote the associated score between *p_i_* and *l_j_*:

(25) $$S_{F}\left(i\right)=\sum_{j=1}^{n}\frac{a_{ij}S_{L}\left(l_{j}\right)}{d\left(l_{j}\right)}=\sum_{j=1}^{n}\frac{a_{ij}}{d\left(l_{j}\right)}\sum_{k=1}^{m}\frac{a_{kj}S_{0}\left(k\right)}{d\left(p_{k}\right)}$$

where $d\left(l_{i}\right)=\sum_{i=0}^{m}a_{ij}$ is the number of proteins interacting with *l_j_*.

Step 5 Computing the final associated score *S_F_* after the above two-step information propagation yields

(26) $${\vec{S}}_{F}=W\vec{S_{0}}$$

where ${\vec{S}}_{0}$ denotes the column vector of *S*_0_, $S_{F}\left(i\right)=\sum_{k=1}^{m}w_{ik}S_{0}\left(k\right)$, where $\mathrm{wij}=\frac{1}{d(pi)}\sum_{j=1}^{n}\frac{a_{ij}a_{kj}}{d(lj)}$.

**Figure 5:**
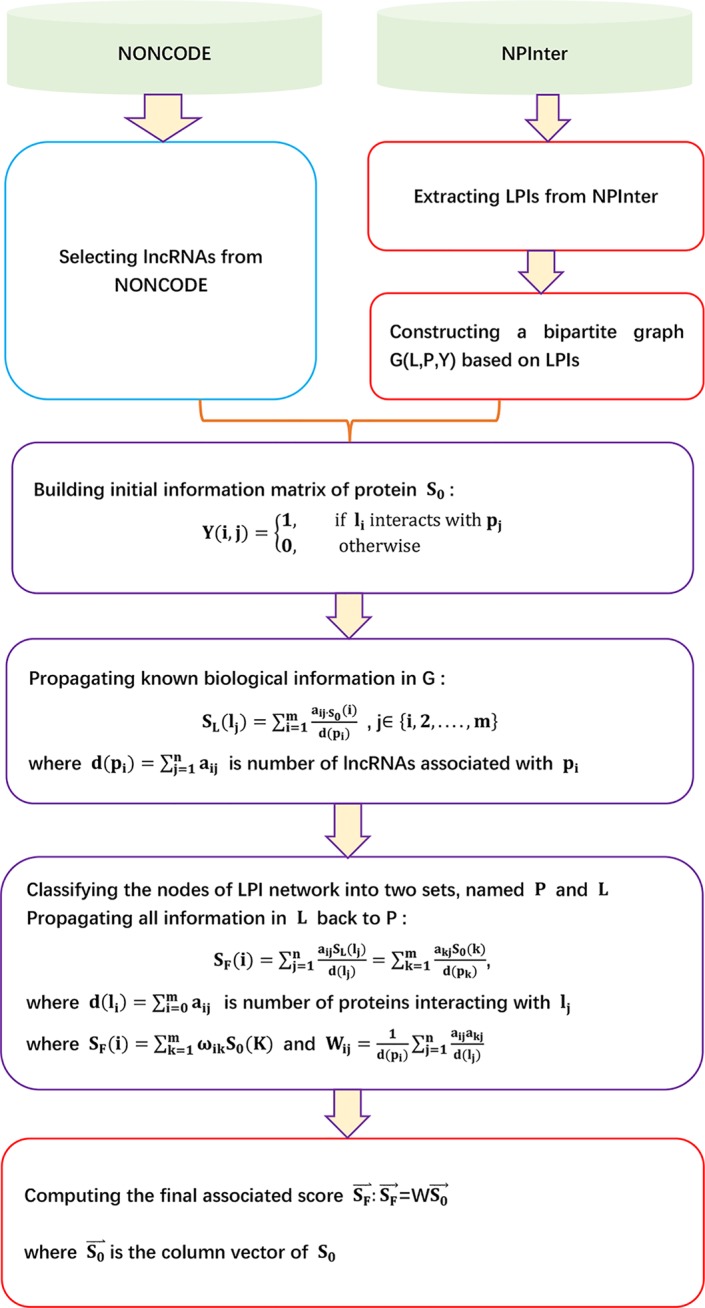
Flowchart of lncRNA–protein bipartite network inference method.

The details are shown in **Figure 5**.

#### ACCBN

Zhu et al. (2019) exploited an ant-colony-clustering-based bipartite network method for revealing potential LPIs, ACCBN. The model can be roughly broken down into three steps.

Step 1 Describing lncRNA interaction profiles and protein interaction profiles as row vectors and column vectors based on the LPI network, respectively.

Step 2 Calculating the probability that two entities *x_i_* and *x_j_* belong to the same cluster based on the ant colony clustering method:

(27) $$p_{ij}\left(t\right)=\frac{{\left[T_{ij}\left(t\right)\right]}^{\alpha }{\left[\eta_{ij}\left(t\right)\right]}^{\beta }}{\sum_{j=1}^{k}{\left[T_{ij}\left(t\right)\right]}^{\alpha }{\left[\eta_{ij}\left(t\right)\right]}^{\beta }}$$

where

(28) $$\eta_{ij}=\frac{1}{d_{ij}}$$

(29) $$d_{ij}={\left(\sum_{k=1}^{m}|x_{ik}-x_{jk}{|}^{2}\right)}^{\frac{1}{2}}$$

(30) $$T_{ij}\left(t+1\right)=\left(1-\rho \right)T_{ij}\left(t\right)+\Delta T_{ij}\left(t\right)$$

(31) $$T_{ij}\left(t\right)=\{\begin{array}{c} 1\;d_{ij}\leq r\\ 0\;d_{ij}>r \end{array}$$

(32) $$\Delta T_{ij}\left(t\right)=\frac{Q}{d\left(x_{i},c_{j}\right)}$$

where *r* is the cluster radius, *c_j_* is the cluster center of the *j*th cluster, and α ∈ (0, 5), *β* ∈ (0, 5), *ρ* ∈ (0.1, 0.99), and *Q* ∈ (1, 10000).

Step 3 Applying lncRNA–protein bipartite network to identify LPI candidates. Given a protein *p_k_*, its association scores with all lncRNAs at the *t*th iteration $P_{k}^{t}$ can be computed as follows:

(33) $$P_{k}^{t}=\rho WP_{k}^{t-1}+\left(1-\rho \right)M\left(:,k\right)$$

where *W* is a similarity matrix.

The association scores for all proteins {*p*_1_, *p*_2_,…, *pm*} can be represented as follows:

(34) $$P^{t}=\rho WP^{t-1}+\left(1-\rho \right)M$$

### Machine Learning-Based Methods

Machine learning-based LPI prediction methods utilize machine learning-based models and algorithms to uncover potential LPIs. This type of method can be roughly classified into two categories: matrix factorization-based methods and ensemble learning-based methods.

#### Matrix Factorization-Based Models

Matrix factorization is exploited in recommendation systems and has been widely applied to bioinformatics (Shi et al., 2018; Zhang et al., 2018a; Zhao et al., 2018b; Cantini et al., 2019). Matrix factorization-based LPI prediction techniques transformed the problem of LPI identification into a recommender task, and adopted the matrix factorization model to capture unobserved LPIs. Given an LPI matrix *Y* and two nonnegative matrices *W* ∈ ℜ*^k^* ^x^ *^n^* and *H* ∈ ℜ*^k^* ^x^ *^m^* the problem of predicting LPIs can be formulated as the following objective function:

(35) $$\underset{W,H}{\min\;}\|Y-W^{T}H{\|}_{F}^{2}\text{ s.t. }W\geq 0,H\geq 0$$

A few LPI identification methods have been designed based on matrix factorization method.

##### LPGNMF

Zhang et al. (2018a) designed a graph regularized nonnegative matrix factorization-based (NMF) method to predict potential LPIs, LPGNMF. LPGNMF consists of three steps.

Step 1 Extracting LPI information based on data provided by NONCODE (Zhao et al., 2015), NPInter (Hao et al., 2016), and UniProt (Consortium et al., 2018). Obtaining 9,484 LPIs between 50 proteins and 2,190 lncRNAs after filtering and removing lncRNAs/proteins only interacting with one protein/lncRNA.

Step 2 Computing lncRNA similarity and protein similarity.

LPGNMF computes the lncRNA expression profile similarity *S^l^* (*i*, *j*):

Given the expression profiles of two lncRNAs *E*_1_ and *E*_2_, LPIHN calculates lncRNA expression similarity based on the Pearson correlation coefficient:

(36) $$S^{l}\left(i,j\right)=\left|\frac{\mathrm{cov}(E_{1},E_{2})}{\sigma_{e_{1}}\sigma_{e_{2}}}\right|$$

where *cov*(*E*_1_, *E*_2_) is the covariance of *E*_1_ and *E*_2_, and $\sigma_{e_{1}}$ and $\sigma_{e_{2}}$ are the standard deviations of *E*_1_ and *E*_2_, respectively.

LPGNMF computes the weight matrix based on lncRNA similarity:

(37) $$M_{ij}^{l}=\{\begin{array}{c} 1\;i\in N\left(l_{j}\right)\;\&\;j\in N\left(l_{i}\right)\\ 0\;i\notin N\left(l_{j}\right)\;\&\;j\notin N\left(l_{i}\right)\\ 0.5\;\mathrm{otherwise} \end{array}$$

Here, *N*(*l_i_*) and *N*(*l_j_*) denote the *p* nearest neighbors of *l_i_* and *l_j_*.

LPGNMF then calculates the sparse similarity matrix of lncRNAs S^l*^:

(38) $$S_{ij}^{l*}=M_{ij}^{l}S_{ij}^{l}$$

Similarly, LPGNMF calculates the sparse similarity matrix of proteins *S^p^* ^*^.

Step 3 Building the following optimization model based on the graph regularized nonnegative matrix factorization method:

(39) $$\underset{W,H}{\min}\|Y-W^{T}H{\|}_{F}^{2}+\lambda_{p}\sum_{i,j=1}^{n}\|w_{i}-w_{j}{\|}^{2}S_{ij}^{p*}+\lambda_{l}\sum_{i,j=1}^{m}\|h_{i}-h_{j}{\|}^{2}S_{ij}^{l*}+\beta_{1}\sum_{i,j=1}^{n}\|W(:,i){\|}_{1}^{2}+\beta_{2}\sum_{i,j=1}^{m}\|H(:,i){\|}_{1}^{2}s.t.\;W\geq 0,H\geq 0$$

**Figure 6:**
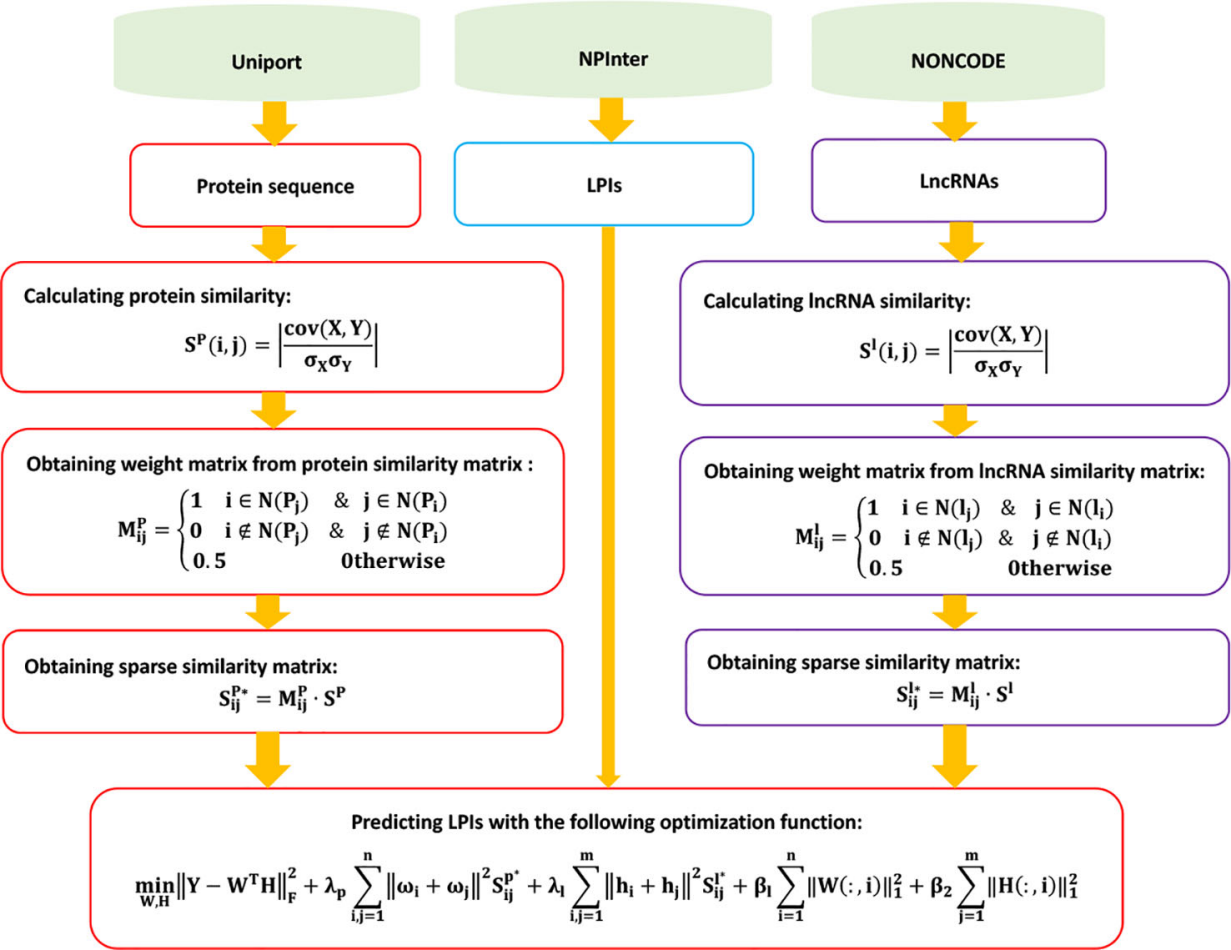
Flowchart of LPI prediction method based on graph regularized nonnegative matrix factorization.

The details are shown in **Figure 6**.

##### LPI-NRLMF

Liu et al. (2017) designed a novel LPI identification model based on neighborhood regularized logistic matrix factorization, LPI-NRLMF. LPI-NRLMF can be roughly broken down into three steps.

Step 1 Extracting the lncRNA sequence, protein sequence, and LPIs based on data provided by NONCODE (Zhao et al., 2015), NPInter (Hao et al., 2016), and UniProt (Consortium et al., 2018); and obtaining 4,158 LPIs between 27 proteins and 990 lncRNAs.

Step 2 Computing lncRNA sequence similarity matrix *LSM* and protein sequence similarity matrix *PSM* based on the Smith–Waterman algorithm:

(40) $$LSM\left(l_{i},l_{j}\right)=\frac{\mathrm{sw}\left(l_{i},l_{j}\right)}{\max\;(\mathrm{sw}\left(l_{i},l_{i}\right),\mathrm{sw}\left(l_{j},l_{j}\right))}$$

(41) $$PSM\left(p_{i},p_{j}\right)=\frac{sw\left(p_{i},p_{j}\right)}{\max\;(sw\left(p_{i},p_{i}\right),sw\left(p_{j},p_{j}\right))}$$

Step 3 Defining neighborhood information for lncRNAs and obtaining the adjacency matrix *A* of lncRNAs:

(42) $$a_{iu}=\{\begin{array}{c} s_{iu}^{l}\;if\;l_{u}\in N\left(l_{i}\right)\\ 0\;otherwise \end{array}$$

Similarly, LPI-NRLMF computes the adjacency matrix *B* of proteins.

Step 4 Computing associated scores *S_N_* for unknown lncRNA–protein pairs based on the neighborhood regularized logistic matrix factorization model:

(43) $$p_{ij}=\frac{exp\;(u_{i}v_{j}^{T})}{1+exp\;(u_{i}v_{j}^{T})}$$

Here, *u_i_* ∈ ℜ^1^ ^x^ *^r^* and *v_j_* ∈ ℜ^1^ ^x^ *^r^* can be computed by the following neighborhood regularized logistic matrix factorization model:

(44) $$\underset{U,V}{\min}\sum_{i=1}^{m}\sum_{j=1}^{n}(1+cy_{ij}-y_{ij})\ln[1+\exp(u_{i}v_{j}^{T})]-cy_{ij}u_{i}v_{j}^{T}+\frac{1}{2}tr[U^{T}(\lambda_{l}I+\alpha L^{l})U]+\frac{1}{2}tr[V^{T}(\lambda_{p}I+\beta L^{p})V]$$

where $L^{l}=\left(D_{i}^{l}+D_{u}^{l}\right)-\left(A+A^{T}\right)$, $D_{i}^{l}=\sum_{u=1}^{m}a_{iu},D_{u}^{l}=\sum_{i=1}^{m}a_{iu}$. Similarly, *L_P_* can be computed. *U* ∈ ℜ*^m^* ^x^ *^r^* and *V* ∈ ℜ^1^ *^x^^r^* can be calculated by dividing *L*.

**Figure 7:**
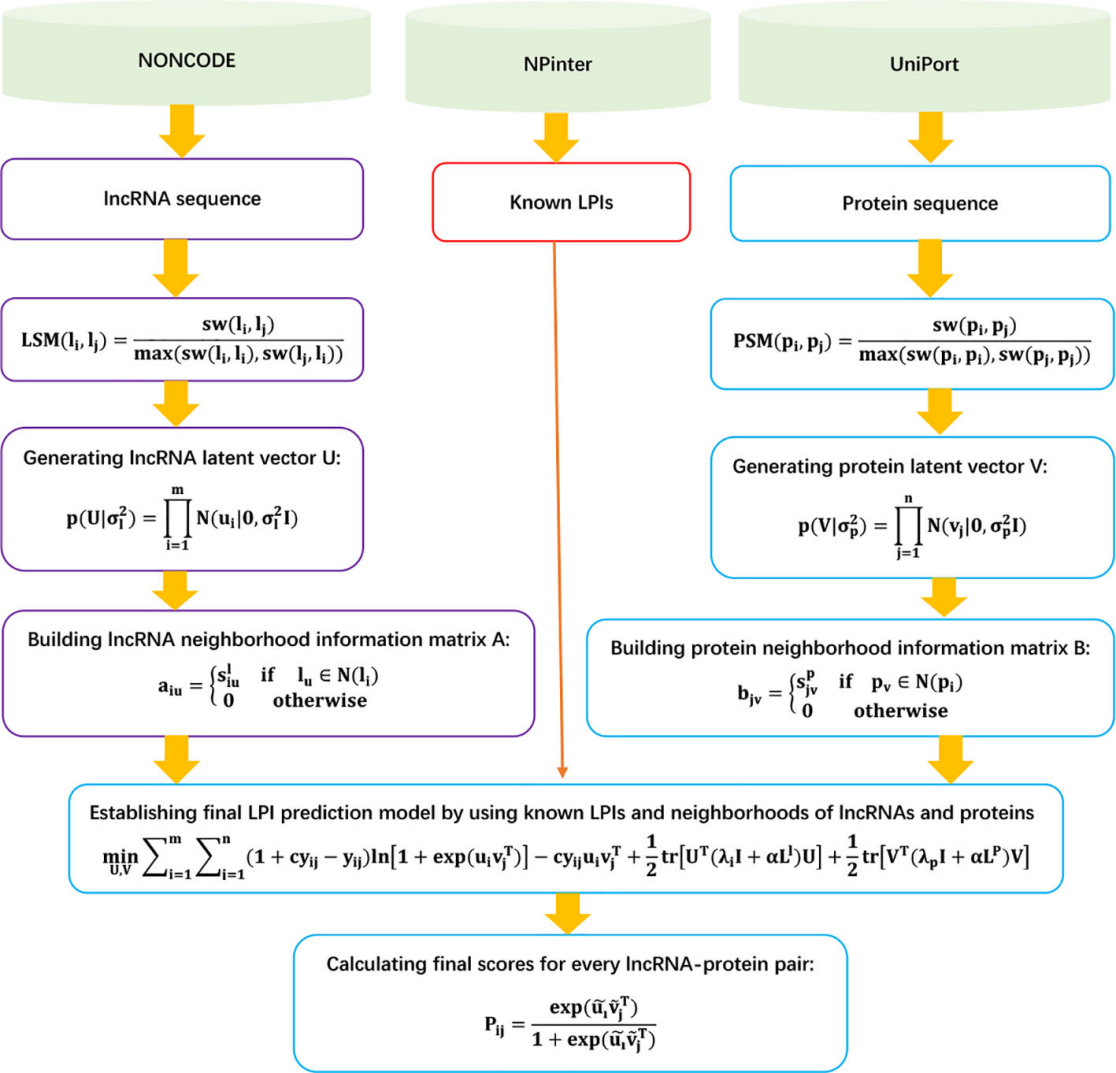
Flowchart of LPI prediction model based on neighborhood regularized logistic matrix factorization.

The details are shown in **Figure 7**.

##### IRWNRLPI

Zhao et al. (2018b) fused the random walk into LPI-NRLMF and exploited a novel LPI prediction model based on LPI-NRLMF, IRWNRLPI. IRWNRLPI is a semi-supervised learning-based model and does not require negative samples. IRWNRLPI contains the following five steps.

Step 1 Extracting the lncRNA sequence, protein sequence, and LPIs from NONCODE (Zhao et al., 2015), NPInter (Hao et al., 2016), and UniProt (Consortium et al., 2018); and obtaining 4,158 LPIs between 27 proteins and 990 lncRNAs.

Step 2 Computing the lncRNA sequence similarity matrix *LS* and protein sequence similarity matrix *PS* based on the Smith–Waterman algorithm:

(45) $$LS\left(l_{i},l_{j}\right)=\frac{sw\left(l_{i},l_{j}\right)}{\max\;(sw\left(l_{i},l_{i}\right),sw\left(l_{j},l_{j}\right))}$$

(46) $$PS\left(p_{i},p_{j}\right)=\frac{sw\left(p_{i},p_{j}\right)}{\max\;(sw\left(p_{i},p_{i}\right),sw\left(p_{j},p_{j}\right))}$$

Step 3 Building a random walk model to compute associated scores *S_R_* for unknown lncRNA–protein pairs:

(47) $$\begin{array}{l} S\left(t+1\right)=r_{Q}L_{Q}^{T}S(t)+p_{Q}\left(1-r_{Q}\right)X+r_{U}L_{U}^{T}S(t)+p_{U}\left(1-r_{U}\right)X \end{array}$$

where *r_ij_* represents the extent of association between a neighbor *v_j_* and a protein *p* for a given node *v_i_*. *L*(*l_ij_*)*_M_*_x_ *_M_* is computed by $l_{ij}=r_{ij}/\sum_{j=1}^{N}r_{ij}$. IRWNRLPI divides *L* into two arrays of *L_U_* and *L_Q_*.

Step 4 Computing associated scores *S_N_* for unknown lncRNA–protein pairs based on the neighborhood regularized logistic matrix factorization model:

(48) $$p_{ij}=\frac{exp\;(u_{i}v_{j}^{T})}{1+exp\;(u_{i}v_{j}^{T})}$$

*u_i_* ∈ ℜ^1^ ^x^ *^r^* and *v_j_* ∈ ℜ^1^ ^x^ *^r^* can be computed by the following neighborhood regularized logistic matrix factorization model:

(49) $$\underset{U,V}{\min}\sum_{i=1}^{m}\sum_{j=1}^{n}(1+cy_{ij}-y_{ij})\ln[1+\exp(u_{i}v_{j}^{T})]-cy_{ij}u_{i}v_{j}^{T}+\frac{1}{2}tr[U^{T}(\lambda_{l}I+\alpha L^{l})U]+\frac{1}{2}tr[V^{T}(\lambda_{p}I+\beta L^{p})V]$$

where *U* ∈ ℜ*^m^* ^x^ *^r^* and *V* ∈ ℜ*^n^* ^x^ *^r^*.

Step 5 Computing the final associated scores for unknown lncRNA–protein pairs:

(50) $$S=\frac{S_{R}+S_{N}}{2}$$

**Figure 8:**
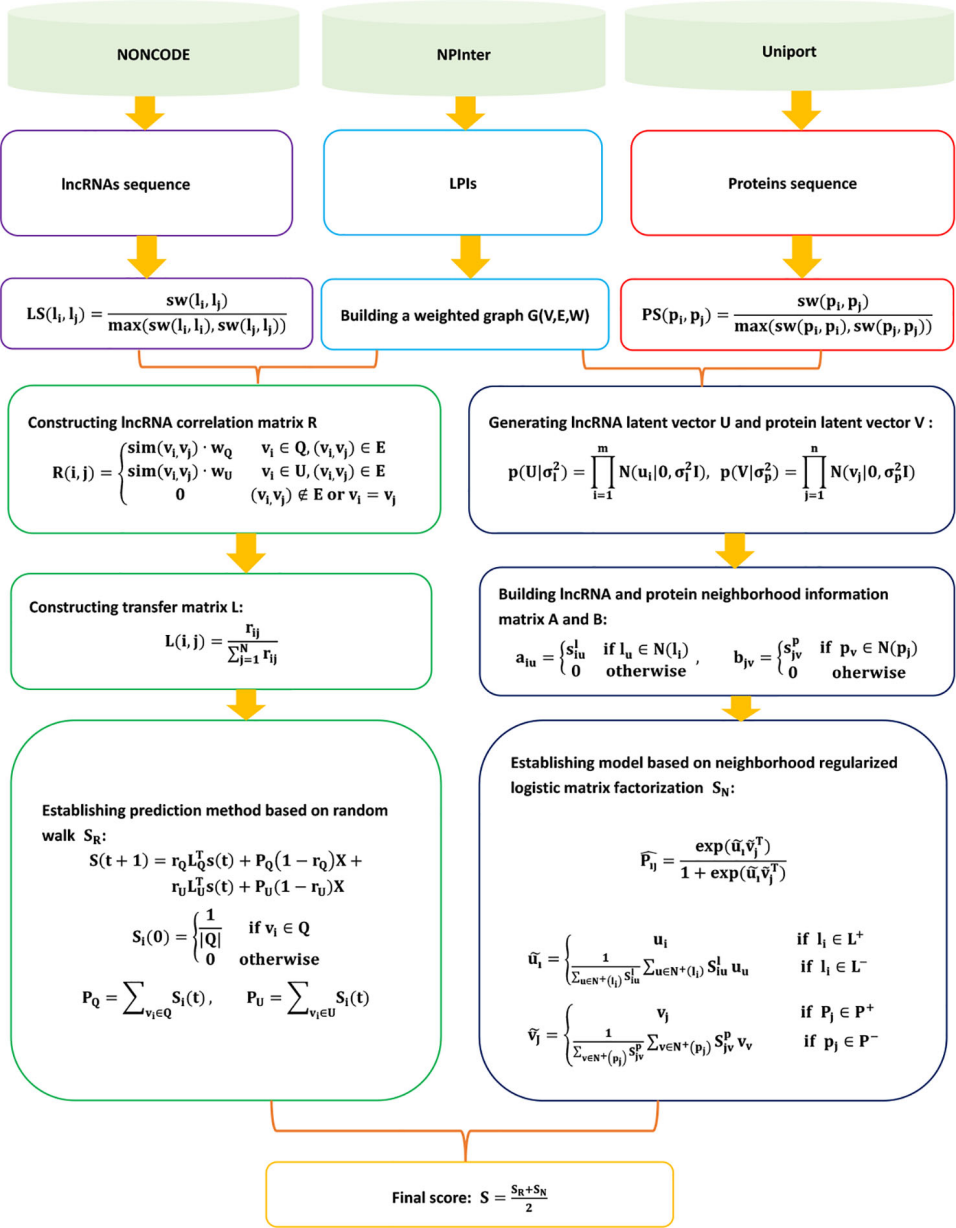
Flowchart of LPI prediction model based on the random walk and neighborhood regularized logistic matrix factorization.

The details are shown in **Figure 8**.

##### LPI-KTASLP

Shen et al. (2019) designed a kernel target alignment-based semi-supervised model, LPI-KTASLP, to find novel LPIs. LPI-KTASLP utilizes matrix factorization and an approximation technique. LPI-KTASLP can be roughly broken down into three steps.

Step 1 Computing lncRNA kernels and protein kernels from four levels.

Level 1 GIP kernel:

The GIP kernels between two lncRNAs and two proteins are defined as follows, respectively:

(51) $$K_{GIP}^{lnc}(l_{i},l_{j})=\exp(-\gamma_{lnc}\|Y_{l_{i}}-Y_{l_{j}}{\|}^{2})$$

(52) $$K_{GIP}^{pro}(p_{i},p_{j})=\exp(-\gamma_{pro}\|Y_{p_{i}}-Y_{p_{j}}{\|}^{2})$$

Level 2 Sequence kernel:

The sequence kernels of two lncRNAs and two proteins are defined as follows, respectively:

(53) $$K_{SW}^{\mathrm{lnc}}(l_{i},l_{k})=\frac{SW(S_{l_{i}},S_{l_{k}})}{\sqrt{SW(S_{l_{i}},S_{l_{i}})}\sqrt{SW(S_{l_{k}},S_{l_{k}})}}$$

(54) $$K_{SW}^{pro}(P_{i},P_{k})=\frac{SW(S_{p_{i}},S_{p_{k}})}{\sqrt{SW(S_{p_{i}},S_{p_{i}})}\sqrt{SW(S_{p_{k}},S_{p_{k}})}}$$

where *SW*(.,.) is the Smith–Waterman score, and *S* represents the sequence information of a lncRNA/protein.

Level 3 Sequence feature kernel:

Constructing radial basis function kernels $K_{SF}^{lnc}$ and $K_{SF}^{pro}$ for lncRNAs and proteins based on the conjoint triad and pseudo position-specific score matrix, respectively.

Level 4 lncRNA expression kernel:

Calculating the expression kernel of lncRNA $K_{EXP}^{lnc}$ based on the expression profiles of lncRNAs provided by the NONCODE database (Zhao et al., 2015).

Step 2 Fusing the above kernels to generate the optimal kernel based on kernel target alignment:

(55) $$K_{lnc}^{*}=\sum_{a=1}^{4}w_{a}^{lnc}K_{a}^{lnc},{\text{K}}_{a}^{lnc}\in {\text{ℜ}}^{n\times n}$$

(56) $$K_{pro}^{*}=\sum_{a=1}^{3}w_{a}^{pro}K_{a}^{pro},{\text{K}}_{a}^{pro}\in {\text{ℜ}}^{m\times m}$$

Step 3 Constructing the following model to compute interaction probabilities for unobserved lncRNA–protein pairs based on matrix factorization, low-rank approximation, and eigen decomposition:

(57) $$Y^{*}=\frac{1}{1+3\delta }Y+\frac{1}{1+3\delta^{2}}V_{lnc}\left(D⊙\left(V_{lnc}^{T}FV_{pro}\right)\right)V_{pro}^{T}$$

**Figure 9:**
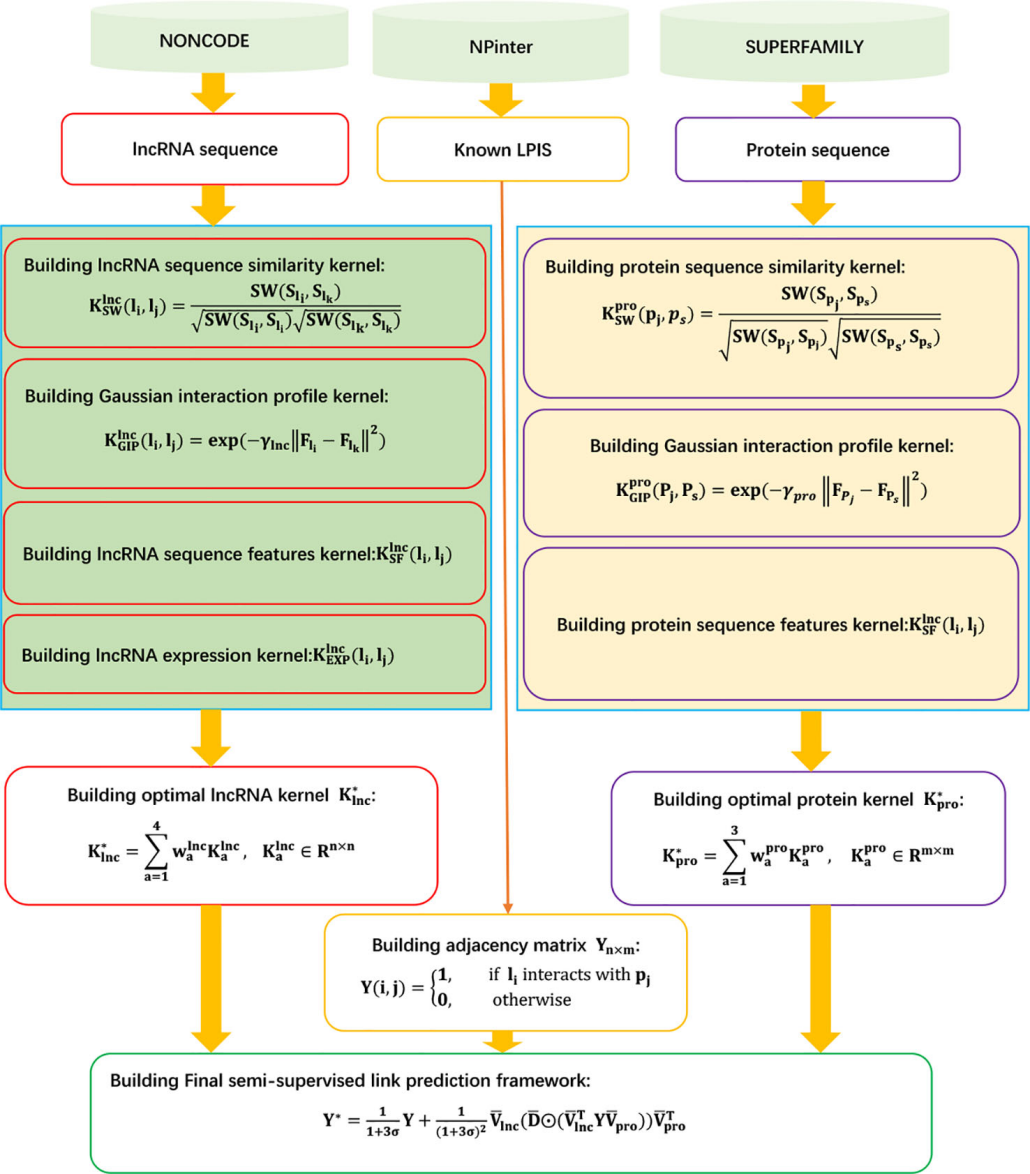
Flowchart of kernel target alignment-based semi-supervised model for LPI prediction.

The details are shown in **Figure 9**.

#### Ensemble-Based Methods

Ensemble learning methods are widely applied to LPI prediction. HLPI-Ensemble (Hu et al., 2018) and SFPEL-LPI (Zhang et al., 2018c) are two state-of-the-art ensemble-based LPI prediction methods.

##### HLPI-Ensemble

Hu et al. (2018) developed the HLPI-Ensemble method for human LPI identification. HLPI-Ensemble consists of two major processes: benchmark dataset construction and HLPI-Ensemble model construction.

In the first process, HLPI-Ensemble downloads lncRNA sequences, protein sequences, and LPIs from NONCODE (Zhao et al., 2015), UniProt (Consortium et al., 2018), and NPinter (Hao et al., 2016). HLPI-Ensemble then extracts 82 features of lncRNAs and 1,516 features of proteins based on Kmer, DAC, and PC-PseDNC-General.

**Figure 10:**
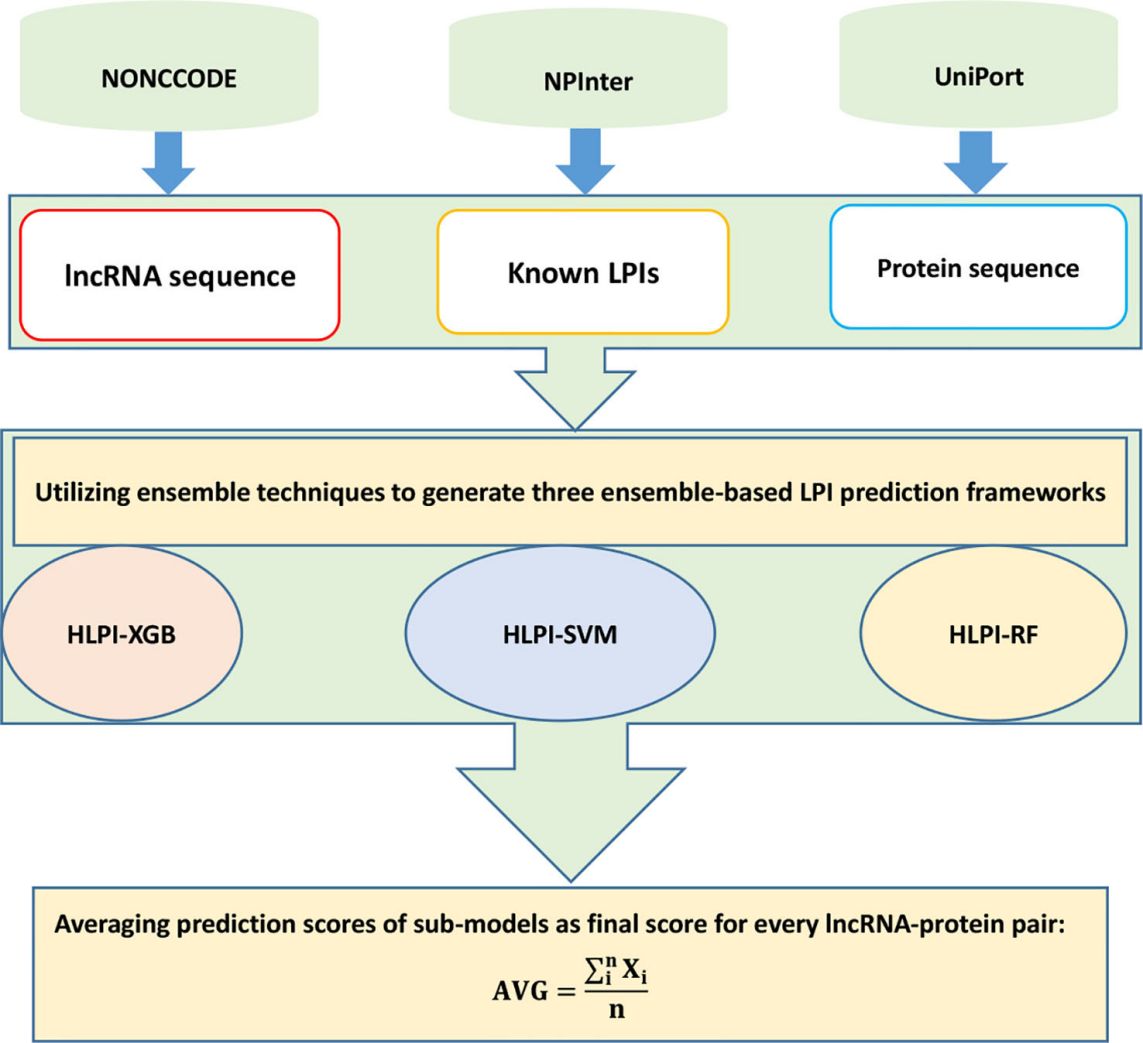
Flowchart of ensemble-based LPI identification method.

In the second process, HLPI-Ensemble utilizes the ensemble technique and generates three ensemble learning frameworks, HLPI-SVM, HLPI-XGB, and HLPI-RF. These three frameworks are based on support vector machines (SVMs), extreme gradient boosting (XGB), and random forests (RFs), respectively. The details are shown in **Figure 10**.

##### SFPEL-LPI

Zhang et al. (2018c) exploited a sequence-based feature projection ensemble learning framework, SFPEL-LPI, to uncover novel LPIs. SFPEL-LPI integrated *ℓ*_1,2_-norm regularization, ensemble graph Laplacian regularization, and various biological information into a unified framework. It can be roughly broken down into five steps.

Step 1 Downloading LPIs, lncRNA sequences, and protein sequences from NPInter (Hao et al., 2016), NONCODE (Zhao et al., 2015), and SUMPERFAMILY (Pandurangan et al., 2018), respectively.

Step 2 Describing lncRNA and protein features based on sequence information and known LPIs.

SFPEL-LPI describes lncRNA features based on parallel correlation pseudo dinucleotide composition (PSEDNC). Given the occurrence frequency of different dinucleotides and the physicochemical properties of every dinucleotide, the PseDNC feature vector for an RNA sequence *L* can be represented as

(58) $$L=[d_{1},d_{2},\ldots \ldots ..d_{16},d_{16+1},\ldots d_{16+\tau }]$$

where

(59) $$d_{k}=\{\begin{array}{cc} \frac{f_{k}}{\sum_{i=1}^{16}f_{i}+w\sum_{j=1}^{\tau }\theta_{j}} & 1\leq k\leq 16\\ \frac{w\theta_{k-16}}{\sum_{i=1}^{16}f_{i}+w\sum_{j=1}^{\tau }\theta_{j}} & 17\leq k\leq 16+\tau \end{array}$$

In addition, SFPEL-LPI represents the interaction profile of an lncRNA as a row vector of the LPI matrix *Y*: $IP_{L_{i}}=Y(i,:)$.

SFPEL-LPI describes protein features based on the parallel correlation pseudo amino acid composition (PseAAC):

(60) $$P=[c_{1},c_{2},\ldots \ldots ..c_{20},c_{20+1},\ldots ,c_{20+\tau }]$$

where

(61) $$c_{k}=\{\begin{array}{cc} \frac{f_{k}}{\sum_{i=1}^{20}f_{i}+w\sum_{j=1}^{\tau }\theta_{j}} & 1\leq k\leq 20\\ \frac{w\theta_{k-20}}{\sum_{i=1}^{20}f_{i}+w\sum_{j=1}^{\tau }\theta_{j}} & 20\leq k\leq 20+\tau \end{array}$$

Similarly, the interaction profile of a protein can be defined as a column vector of the LPI matrix *Y*: $IP_{p_{i}}=Y(:,i)$.

Therefore, *a* features for lncRNAs/proteins can be represented as feature matrix: ${\left\{X_{i}\right\}}_{i=1}^{a}$.

Step 4 Computing lncRNA similarity and protein similarity.

SFPEL-LPI first computes the linear neighborhood similarity of lncRNAs based on PseDNC and IP.

SFPEL-LPI then computes the Smith–Waterman subgraph similarity (SWSS) of lncRNAs:

(62) $$\mathrm{SWSS}(L_{i},L_{j})=\sum_{P_{01}\in A(L_{i})}\sum_{P_{02}\in A(L_{j})}\frac{SW(P_{o1},P_{o2})}{n1\times n2}$$

Similarly, the PseAAC similarity, IP similarity, and SWSS similarity of proteins can be computed.

Therefore, *b* types of similarities of lncRNAs/proteins can be represented as *b* similarity matrices ${\left\{W_{i}\right\}}_{i=1}^{b}$.

Step 5 Computing the association scores for novel lncRNAs/proteins based on Eqs. (63) and (64).

(63) $$R_{l}=\sum_{i=1}^{u}\theta_{li}X_{li}G_{li}^{T}$$

(64) $$R_{p}=\sum_{i=1}^{v}\theta_{pi}X_{pi}G_{pi}^{T}$$

*G_i_*, *R*, and *θ* can be obtained by solving the following optimization model:

(65) $$\begin{array}{l} \underset{G_{i},R,\theta }{\min}\|R-Y{\|}_{F}^{2}+\mu \sum_{i=1}^{a}\|X_{i}G_{i}^{T}-R{\|}_{F}^{2}+\sum_{i=1}^{b}\theta_{i}^{\eta }tr(R^{T}(D_{i}-W_{i})R)+\lambda \sum_{i=1}^{a}\|G_{i}{\|}_{1,2}^{2}\\ s.t.\;G_{i}\geq 0,\sum_{i=1}^{b}\theta_{i}=1 \end{array}$$

**Figure 11:**
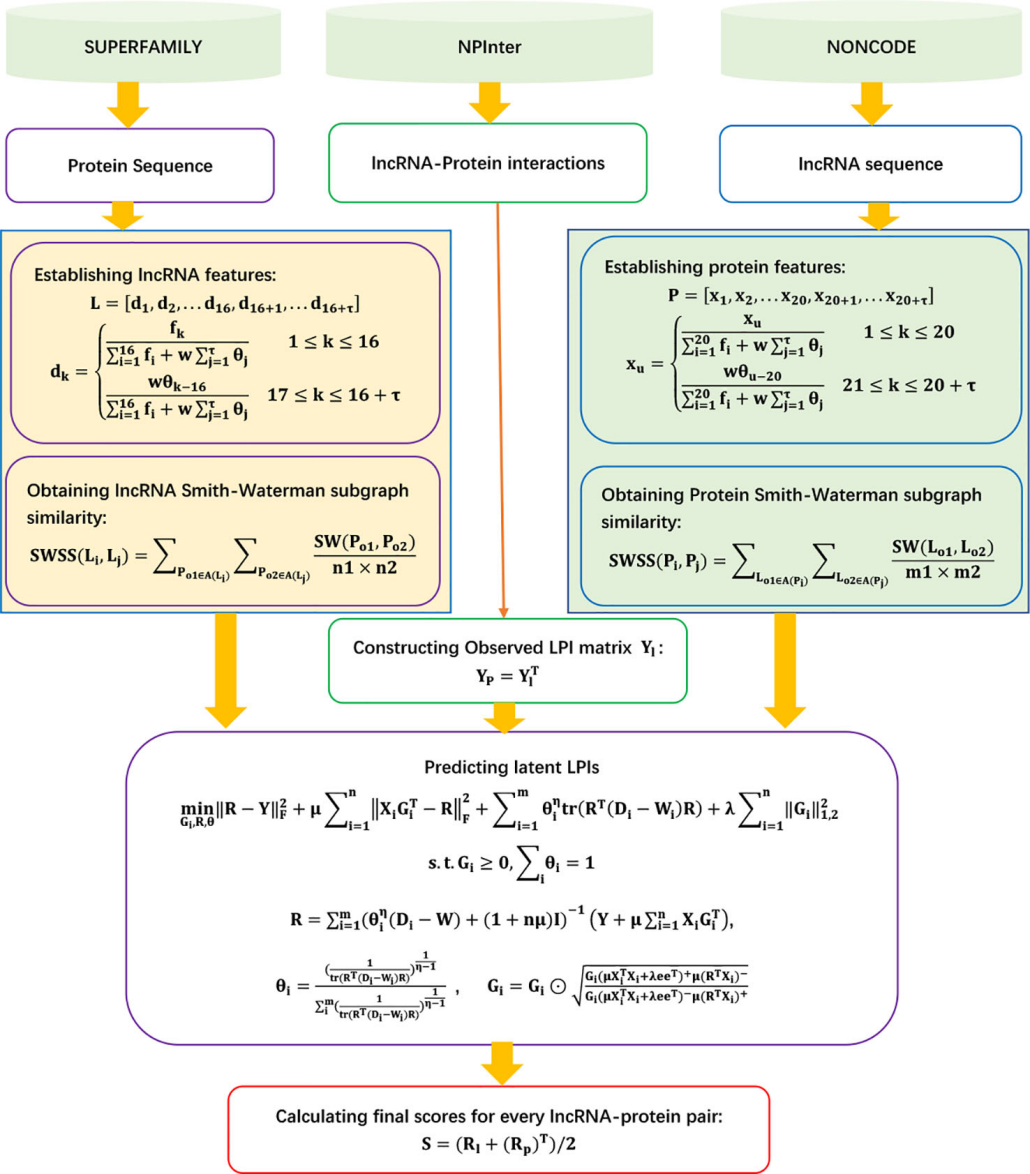
Flowchart of LPI prediction method based on sequence feature projection ensemble learning framework.

The details are shown in **Figure 11**.

##### Other Methods

There are several methods used to predict possible LPIs except for matrix factorization-based methods and ensemble learning-based methods, for example, Fisher's linear discriminant-based LPI prediction method (IncPro) (Lu et al., 2013), eigenvalue transformation-based semi-supervised model (LPI-ETSLP) (Hu et al., 2017), and kernel ridge regression model based on fast kernel learning(LPI-FKLKRR) (Shen et al., 2018).

##### lncPRO

Lu et al. (2013) explored a Fisher's linear discriminant-based LPI prediction method, lncPro. lncPro found new LPI through executing the following four steps.

Step 1 Downloading complexes data from the PDB database.

Step 2 Encoding sequence information into numerical feature vectors for lncRNAs and proteins based on the secondary structure, the Van der Waals' propensities, and the hydrogen-bonding propensities.

Step 3 Transforming the feature vectors to unify the dimension based on the Fourier series:

(66) $$X_{k}^{\prime }=\sqrt{\frac{2}{L}}\sum_{i=0}^{L}X_{i}\cos\left[\frac{\pi }{L}\left(n+\frac{1}{2}\right)\left(k+\frac{1}{2}\right)\right]\;k=0,1,\ldots ,9$$

where *L* is the length of feature vector of lncRNAs/proteins.

Step 4 Calculating the final score matrix < *p*|*M*|*r*> for the RNA feature vector *r* and a protein feature vector *p* based on Fisher's linear discriminant method:

(67) $$<p|M|r>=M_{1}p_{1}r_{1}+M_{2}p_{1}r_{2}+M_{3}p_{2}r_{1}+M_{4}p_{2}r_{2}$$

##### LPI-ETSLP

Hu et al. (2017) presented an eigenvalue transformation-based semi-supervised model, LPI-ESTLP, to uncover the underlying LPIs. LPI-ESTLP can be broken down into three steps.

Step 1 Downloading lncRNA sequences, protein sequences, and LPIs from NONCODE (Zhao et al., 2015), UniProt (Consortium et al., 2018), and NPInter (Hao et al., 2016); and extracting 4,158 LPIs between 27 proteins and 990 lncRNAs after preprocessing.

Step 2 Computing the lncRNA sequence similarity matrix *LSM* and protein sequence similarity matrix *PSM* based on the Smith–Waterman algorithm:

(68) $$LSM(l(i),l(j))=\frac{sw(l(i),l(j))}{\max(sw(l(i),l(i)),sw(l(j),l(j)))}$$

(69) $$PSM(p(i),p(j))=\frac{sw(p(i),p(j))}{\max(sw(p(i),p(i)),sw(p(j),p(j)))}$$

Step 3 Calculating the score matrix based on the following objective function:

(70) $$\bar{Y}=\frac{{\bar{Y}}_{l}+{\bar{Y}}_{p}}{2}$$

where

(71) $$\begin{array}{l} {\bar{Y}}_{l}=(\sigma L_{l}+I{)}^{-1}Y\\ {\bar{Y}}_{p}=(\sigma L_{p}+I{)}^{-1}Y \end{array}$$

and *L_l_* = *I* – *LSM* and *L_p_* = *I* – *PSM* denote the Laplacian matrices of lncRNAs and proteins, respectively.

LPI-ETSLP can obtain the final scores between unobserved lncRNA–protein pairs by integrating eigenvalue transformation into Eq. 70:

(72) $$\bar{Y}=\frac{1}{2}(V_{l}{\bar{U}}_{l}V_{l}^{T}+V_{p}^{T}{\bar{U}}_{p}V_{p})$$

where *Ū_l_* is a diagonal matrix with ${\left[{\bar{U}}_{l}\right]}_{ii}={\left(1+\sigma \left(1-\lambda_{l_{i}}^{\alpha }\right)\right)}^{-1}$. *L_l_* = *I* – *D_l_* ^–0.5^ *K_l_ D_l_* ^–0.5^ and the eigen decomposition of *K_l_* can be expressed as $\bar{K_{l}}=\bar{V_{l}}U_{l}{\bar{\;V}}_{l}$. Similarly, $\bar{K_{p}}=\bar{V_{p}}U_{p}{\bar{\;V}}_{p}$ and ${\bar{\;U}}_{p}$ can be defined.

**Figure 12:**
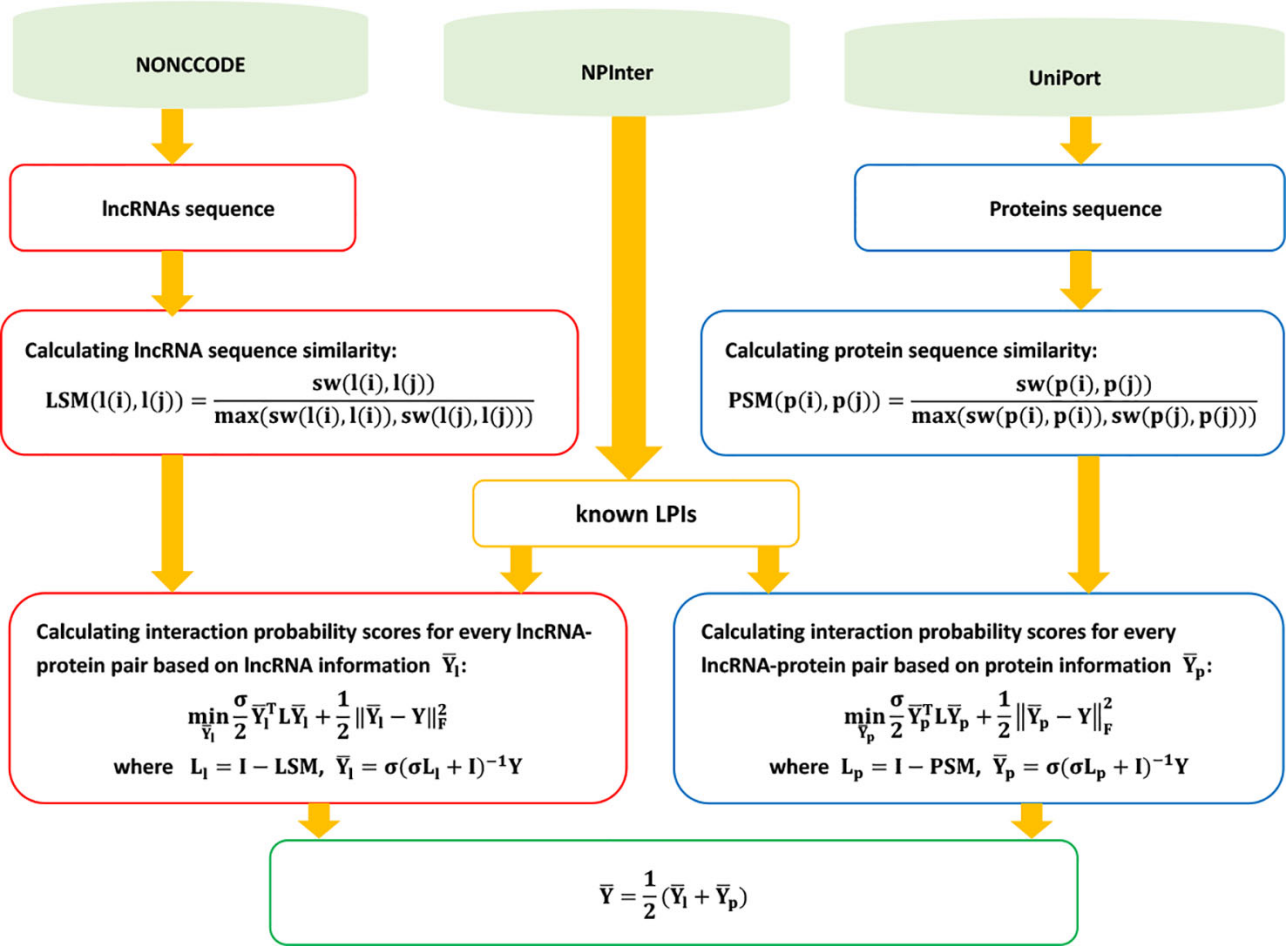
Flowchart of eigenvalue transformation-based semi-supervised model.

The details are shown in **Figure 12**.

##### LPI-FKLKRR

Shen et al. (2018) developed an LPI prediction algorithm, LPI-FKLKRR, combining a kernel ridge regression model based on fast kernel learning. LPI-FKLKRR can be broken into six steps:

Step 1 Computing lncRNA GIP, sequence feature, sequence similarity, and lncRNA expression kernels $K_{GIP}^{lnc}$, $K_{SW}^{lnc}$, $K_{SF}^{lnc}$, and $K_{EXP}^{lnc}$.

Step 2 Computing protein GIP, sequence features, protein sequence similarity, and protein GO kernel $K_{GIP}^{pro}$, $K_{SW}^{pro}$, $K_{SF}^{pro}$, $K_{GO}^{pro}$.

Step 3 Generating the optimal lncRNA and protein kernels with fast kernel learning:

(73) $$K_{lnc}=\sum_{a=1}^{4}w_{a}^{lnc}K_{a}^{lnc},K_{a}^{lnc}\in {ℜ}^{m\times m}$$

$$K_{pro}=\sum_{a=1}^{4}w_{a}^{pro}K_{a}^{pro},K_{a}^{pro}\in {ℜ}^{m\times m}$$

where $w_{a}^{lnc}$ and $w_{a}^{pro}$ represent each element in *w*_*lnc*_ and *w*_*pro*_, respectively; $K_{a}^{lnc}$ and $K_{a}^{pro}$ denote the corresponding normalized similarity matrices in lncRNA and protein spaces, respectively.

Step 4 Constructing the optimization model to compute the optimal solution for *w^lnc^* or *w^pro^*:

(74) $$\begin{array}{l} \underset{w}{\min}\;w^{T}(A+\lambda I)w-2b^{T}w\\ s.t.\sum_{a}^{J}w_{a}=1\\ A_{u,v}=tr(K_{u}^{T}K_{v}) \end{array}$$

where *w* denotes the optimal solution *w_lnc_* or *w_pro_*, *K_u_* and *K_v_* denote two different kernel matrices, and *tr*(·) denotes the trace function.

Step 5 Computing lncRNA–protein association score matrix:

(75) $$F^{*}=K_{lnc}{\left(K_{lnc}+\lambda_{\ell }I\right)}^{-1}F{\left(K_{pro}+\lambda_{p}I\right)}^{-1}K_{pro}$$

Step 6 Producing the optimal F* by adjusting the parameters λ_ℓ_ and λ*_p_*.

**Figure 13:**
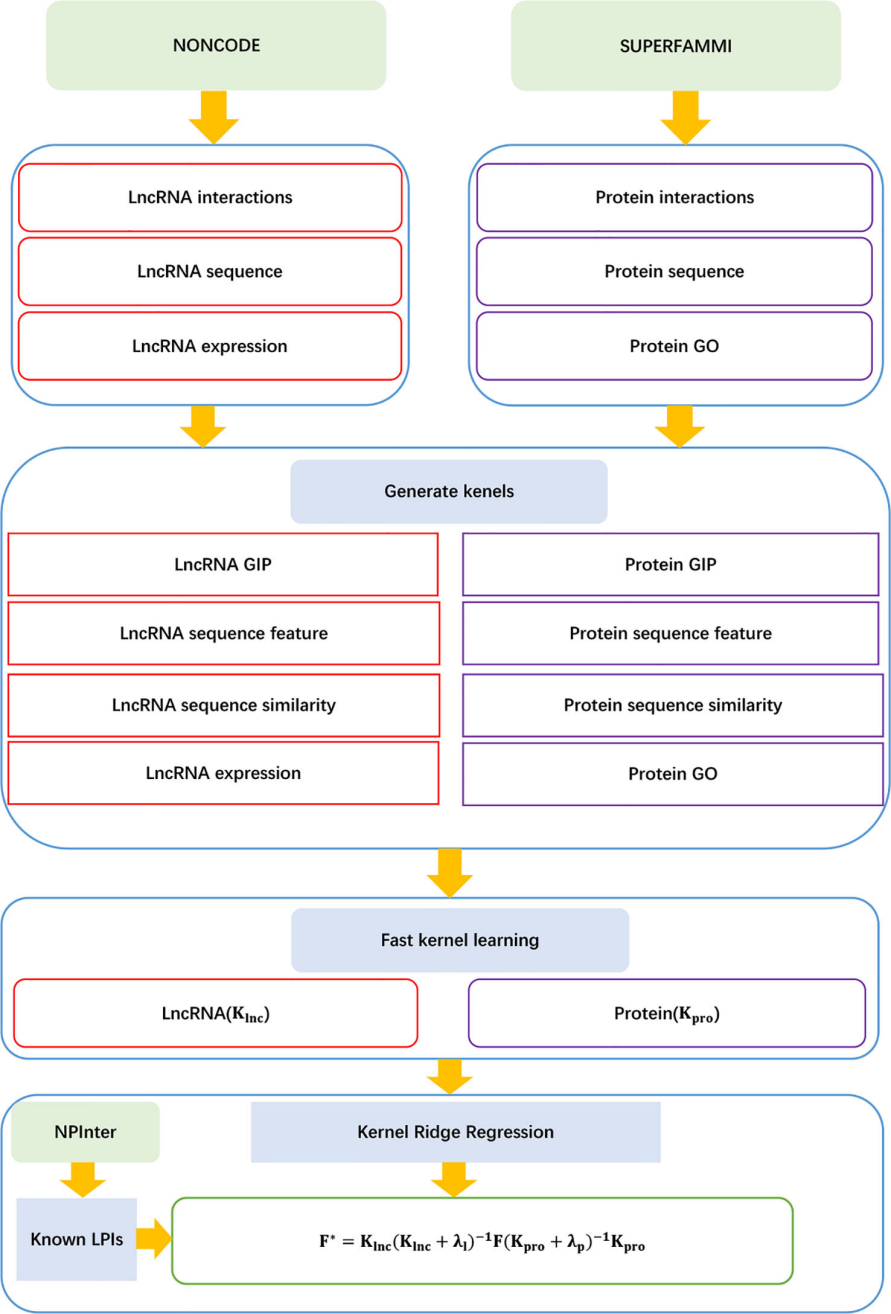
Flowchart of LPI prediction method based on fast kernel learning with kernel ridge regression.

The details are shown in **Figure 13**.

## Discussion

lncRNAs play important regulatory roles in diverse biological processes, such as protein modification, DNA methylation, and chromosome (Weber et al., 2018; Huang et al., 2018a; He et al., 2018b; Zhao et al., 2018c). However, the regulatory mechanism remains unknown (Esteller, 2011; Jiang et al., 2018; Agirre et al., 2019). Studies reported that identifying protein molecules binding specific lncRNAs help to probe the mechanism of lncRNAs (Lu et al., 2013; Ge et al., 2016; Chen et al., 2018). Therefore, identifying possible LPIs has an important role in understanding lncRNA-related activities (Lu et al., 2013; Pan et al., 2016; Peng et al., 2017; Zhang et al., 2018c).

However, experimental methods are expensive and time-consuming. For limited existing knowledge, computational methods become vital as a silver-bullet solution to capture LPIs on a large scale, which contributes to prioritize LPI candidates and deploys further experimental validation (Chen et al., 2018).

In this study, databases involved in LPI identification are summarized. More importantly, the components of state-of-the-art computational models for LPI prediction, such as network-based methods and machine learning-based methods, are introduced. Particularly, machine learning-based models can be broken into matrix factorization-based methods and ensemble learning-based methods. To consider the performance of LPI prediction methods, we compared nine models (IRWNRLPI, LPBNI, LPGNMF, LPI-BNPRA, LPI-ETSLP, LPIHN, LPI-NRLMF, LPLNP, and SFPEL-LPI) on leave-one-out cross-validation (LOOCV). These nine models are conducted on the datasets provided by the corresponding papers. Parameters are set as the values recommended by the corresponding studies. **Table 1** shows the comparison results based on AUC, precision, accuracy, and F1. In **Table 1**, SFPEL-LPI obtained the best performances of AUC and accuracy; LPGNMF obtained the best performances of precision and F1. The results demonstrated that SFPEL-LPI can correctly predict LPIs with a relative high proportion. LPGNMF can better identify potential LPIs when taking into account the proportion of correctly predicted LPIs and successfully predicted LPIs.

**Table 1:** Performance of LPI prediction methods on LOOCV.

Methods	AUC	precision	accuracy	F1
IRWNRLPI	0.9150	0.7178	0.9009	0.6516
LPBNI	0.8586	0.9681	0.9581	0.3868
LPGNMF	0.8520	**1**	0.7854	**0.6871**
LPI-BNPRA	0.8754	0.6540	0.8799	0.5564
LPI-ETSLP	0.8876	0.5932	0.8834	0.5978
LPIHN	0.8030	0.3713	0.9581	0.3868
LPI-NRLMF	0.9025	0.6129	0.8804	0.6197
LPLNP	0.9594	0.1153	0.9592	0.1621
SFPEL-LPI	**0.9735**	0.0016	**0.9731**	0.0033

These bolded texts represent that the corresponding method is the best among comparison methods.

To further detect the performance of SFPEL-LPI, we compared it with four representative LPI prediction methods, LPBNI, LPI-ETSLP, LPIHN, and LPLNP, on fivefold cross-validation. The experiments were conducted on the same dataset, i.e., LPIs, lncRNA sequences, and protein sequences are from NPInter (Hao et al., 2016), NONCODE (Zhao et al., 2015), and SUMPERFAMILY (Pandurangan et al., 2018), respectively. The details are shown in **Table 2**. The results demonstrate that SFPEL-LPI obtained the best performance of AUC and can better identify possible LPIs.

**Table 2:** Performance of LPI prediction methods on fivefold cross-validation.

Methods	AUC	Precision	Accuracy	F1
LPBNI	0.84177	0.2898	0.9431	0.3336
LPI-ETSLP	0.8876	**0.5932**	0.8834	**0.5978**
LPIHN	0.8531	0.4139	0.9581	0.3868
LPLNP	0.9104	0.4102	**0.9646**	0.4520
SFPEL-LPI	**0.9200**	0.4490	0.9600	0.4702

These bolded texts represent that the corresponding method is the best among comparison methods.

In general, network-based methods have become one type of effective tool in possible LPI identification by utilizing LPI network, lncRNA similarity network, and protein similarity matrix. Although network-based methods efficiently discovered unknown LPIs and obtained promising results from the perspective of propagation (Li et al., 2015; Ge et al., 2016; Zheng et al., 2017; Zhao et al., 2018b), this type of method has some weaknesses.

1. Parts of computational methods tested their performances only on one database, which may result in biased predictions because of the sparse nature of LPI data (Li et al., 2015). More importantly, the lack of known LPIs limits the further research of LPI prediction in a larger network (Ge et al., 2016).
2. It is important to unravel potential LPIs for lncRNAs/proteins without any associated information (we represent these lncRNAs/proteins as new lncRNAs/proteins); however, most network-based models fail to capture LPI candidates (Zhang et al., 2018b).
3. Current network-based methods tend to be biased to the lncRNAs/proteins with more known associated proteins. Some lncRNAs/proteins interact with multiple proteins/lncRNAs and others interact with a few or even only one protein/lncRNA in an LPI network. The unbalanced nature of degree distributions in the LPI network may affect prediction performance. Increasing resistance based on the random walk may improve predictive accuracy for LPI prediction models (Li et al., 2015).
4. Parts of methods compute lncRNA similarities based on the expression profile and may produce incomplete coverage of the lncRNA similarity network when adding LPI datasets. This problem may be solved by increasing appropriate data including LPIs (Li et al., 2015).
5. Network-based methods can be applied to an LPI network in which there exists at least one link between two nodes. Especially for a bipartite network, network-based methods require that each node in the network has at least two linkages. However, the LPI network is usually composed of a few isolated subnetworks, and most of the existing network-based models fail to identify the LPIs between the lncRNAs in one subnetwork and the proteins in another (Ge et al., 2016).
6. Most current network-based methods utilized local network information and showed better performance; however, many previous computational biology studies showed that global network information contributes to capturing the associations between two entities, such as LPIs (Karuza et al., 2016; Meng et al., 2016; Shi et al., 2017).
7. Biology finally aims at providing personalized medicine for cancer patients, and it is a key issue to predict relevant drugs/targets for a certain disease by integrating multiple heterogeneous networks and constructing multiple-partite biological networks, such as protein–lncRNA–disease association networks and drug–protein–lncRNA–disease networks. However, current network-based methods are still not applied to this type of prediction (Yao et al., 2016; Yang et al., 2017; Bester et al., 2018; Lu et al., 2018; Ping et al., 2018; Fan et al., 2019).

In summary, machine learning-based LPI prediction methods have some limitations.

1. There are no non-LPIs (negative samples) with experimental validation; therefore, most supervised learning-based LPI prediction models can only randomly select unknown lncRNA–protein pairs as negative LPIs. However, this part of randomly selected negative LPIs may contain true LPIs (positive samples) as well, which significantly influences the predictive performance (Liu et al., 2017; Zhao et al., 2018a; Zhao et al., 2018b; Zhang et al., 2018c; Shen et al., 2019). Although semi-supervised learning-based models utilized unlabeled information to decrease the limitations of negative LPI selection, it still has the same disadvantage as classifier combination (Liu et al., 2017; Zhang et al., 2018a; Shen et al., 2019).
2. Some machine learning-based methods constructed two different classifiers, based on lncRNAs and proteins, respectively. The final results are an average of the performances of two predictive models. This type of model will produce biased results (Zhao et al., 2018b).
3. Many lncRNAs/proteins do not have known association information with any proteins/lncRNAs, and we represent them as new lncRNAs/proteins. Most current predictive models are unable to capture possible proteins/lncRNAs for new lncRNAs/proteins (Zhang et al., 2018c).
4. The proposed methods rely heavily on known LPI data; however, the current number of known LPIs is still very low. Therefore, most machine learning-based models are trained using RNA–protein interaction information instead of LPI data. This results in limited predictive performances (Liu et al., 2017; Zhao et al., 2018a). With the increase in experimentally validated LPIs, the prediction performances of models will improve (Zhao et al., 2018b).
5. The better performances of existing machine learning methods rely severely on data called features (Goodfellow et al., 2016). Current computational methods utilize various lncRNA features and protein features. However, identifying more appropriate features for a given task is still a challenge (Liu et al., 2017; Min et al., 2017). More importantly, these features are not available for all proteins or lncRNAs (Liu et al., 2017; Zhang et al., 2018c).
6. Most experimental data are provided by the NPInter database. NPInter is a relatively abundant database for lncRNA and protein data, but it only provides gene–protein interaction data corresponding to relevant lncRNAs instead of direct LPIs. Gene–protein interactions were directly applied to machine learning-based methods to find possible ncRNA–protein associations and did not discover true LPIs (Liu et al., 2017; Zhao et al., 2018a; Zhao et al., 2018b).
7. Most current computational models for LPI interaction prediction are measured based on cross-validation. Park and Marcotte (2012) used a proteochemometrics model (Wikberg and Mutulis, 2008) for drug–protein interaction prediction and observed that the paired nature of input samples has significant implications on the cross-validation of these pair-input methods. That is to say, there are significant cross-validation differences between input sample and out-of-sample interactions (Park and Marcotte, 2012). For drug–target interaction identification problems, the paired feature of input samples may produce a natural partition of test pairs, and thus the pair-input methods may obtain significantly distinct prediction accuracies for different test classes (Chen et al., 2015). The same situation applies to LPI prediction, which is still a pair-input computational identification problem.

## Conclusion and Further Research

There are a few LPIs and numerous unknown lncRNA–protein pairs not validated by experimental methods in the existing databases. In addition, similar lncRNAs tend to interact with similar proteins, and vice versa (Xiao et al., 2017; Zhang et al., 2018a). Therefore, LPI data have a sparse, low-rank, and unbalanced nature (Li et al., 2015; Zhang et al., 2018a; Shen et al., 2019). With the development of experimental technology, more LPIs will be confirmed, and thus the prediction accuracy of computational models will increase. In this section, we present some suggestions for further research based on the nature of LPI data.

### Fusing Comprehensive LPI Datasets

Parts of computational methods tested their performances only on one database, which may result in biased predictions because of the sparse nature of LPI data (Li et al., 2015). More importantly, existing computational models utilize various biological information from proteins and lncRNAs, for example, physicochemical properties including hydrogen bonding, secondary structure, and van der Waals propensities (Belluci et al., 2011; Xiao et al., 2017). It is important to utilize diverse biological features to improve the performances of LPI prediction models. However, these features are not available for all proteins or lncRNAs, and thus computational methods cannot capture LPI candidates when information is unavailable (Zhang et al., 2018c). Therefore, exploring advanced data fusion methods to integrate more available data sources may further boost the performance of LPI identification.

Focusing on the drawbacks of current network-based LPI identification methods, future research can begin with integrating more heterogeneous networks, such as protein–protein interaction network (Zhang et al., 2019a), lncRNA–miRNA interaction network (Zeng et al., 2016; Huang et al., 2018c; Zhao et al., 2019), lncRNA–mRNA interaction network (Alaei et al., 2019), lncRNA–disease association network (Fu et al., 2017; Wang et al., 2019), and lncRNA–miRNA–mRNA regulatory network (Chen et al., 2018; Zhang et al., 2019b). However, how to address the data conflict problems while integrating diverse LPI data from different repositories is a challenge.

Although there are not currently data conflict solutions for LPI prediction, we can find some clues by other problems in the area of bioinformatics. For example, Liu et al. (2015) set a confidence level for each DTI and gave a higher score to a DTI from a more reliable data repository. For example, the STITCH database assigns a score with a range [0, 1,000] to each DTI based on four types of different sources: model prediction, text mining, manually curated databases, and experimental validation. Particularly, Liu et al. (2015) gave DTIs from Matador and DrugBank the highest values (1,000) because DTIs from these two databases are reported by biochemical experiments and relevant studies. Lou et al. (2017) exploited another type of data fusion from a multiple-views perspective. This involved five steps: screening relevant information from different data sources; removing isolated nodes without edges in the networks; fusing various types of nodes and edges and building a heterogeneous network; constructing multiple similarity networks to boost the network heterogeneity; and excluding homologous nodes from the constructed heterogeneous networks to further reduce the possible redundancy of associated information. Inspired by these two methods, we can fuse diverse heterogeneous data to improve performance in future research. More importantly, new exploited network-based methods should be implemented on a constructed heterogeneous network rather than a single network.

### Screening Credible Negative Samples

There are some known LPIs (positive samples) and abundant unknown lncRNA–protein pairs in existing LPI data resources. More importantly, there are no experimentally validated non-LPIs, and thus most supervised learning-based models have no other choice but to randomly screen negative LPIs from unlabeled lncRNA–protein pairs or even regarded all unlabeled lncRNA–protein pairs as negative samples (Liu et al., 2017; Zhao et al., 2018b). However, the randomly screened negative LPIs may contain positive LPIs as well, and thus there are severe biases in supervised learning-based techniques. Therefore, exploiting an efficient model to select high-quality negative samples is a challenging task for boosting LPI prediction accuracy.

Cheng et al. (2017) designed a FInding Reliable nEgative samples method (FIRE) to select negative RNA–protein interactions. FIRE was based on the following assumption: given a known RNA–protein interaction between an RNA *i* and a protein *j*, for an RNA *k*, the more differences between *i* and *k*, the less possibility that *k* interacts with *j*, and vice versa. FIRE screened negative RNA–protein interactions through the following steps: computing the protein similarity matrix, building a positive sample set based on known interaction information, scoring an unknown RNA–protein pair not included in positive sample set based on protein similarities, generating *m* negative samples by sorting these RNA–protein pairs *via* their scores in increasing order, and selecting the top-*m* RNA–protein pairs. Similarly, we may generate negative LPIs based on lncRNA–lncRNA similarities, protein–protein similarities, and the above assumption.

Positive-unlabeled (PU) learning (de Campos et al., 2018; Sansone et al., 2018; Yang et al., 2018) is applied to various situations. In PU learning, a supervised learning-based method is designed to learn a classification model from a positive sample set and an unlabeled dataset from an unknown class. Yang et al. (2018) designed an adaptive sampling framework with class label noise based on PU learning and introduced two new bioinformatic applications: identifying kinase-substrates and identifying transcription factor target genes. Therefore, PU learning may be one strong way to solve the problem of lacking negative LPIs.

### Deep Learning

Existing computational methods have utilized different lncRNA features and protein features. For example, Bellucci et al. (2011) integrated three types of physicochemical properties, including hydrogen bonding, secondary structure, and van der Waals propensities; meanwhile, Lu et al. (2013) used six types of RNA secondary structures (besides physicochemical properties), which were provided by Bellucci et al. (2011). Therefore, designing more powerful models to integrate relevant biological features is a key issue. However, features are typically exploited by human biomedical engineers, and determining which features are more suitable for LPI prediction remains difficult. More importantly, encoding vectors that are too short may restrict the prediction accuracy of classification model. More importantly, most computational models only used sequence information but did not consider structure information (Peng et al., 2019).

Deep learning-based computational models composed of multiple processing layers require very little engineering knowledge and can efficiently extract features from raw data and construct high-level representations (Wei et al., 2018; Peng et al., 2019). These types of models have been applied to diverse analysis problems, and have obtained better performance due to the excellent power of feature learning (Jurtz et al., 2017; Min et al., 2017; Peng et al., 2019). Therefore, it is valuable and feasible to exploit deep learning-based methods to highly and effectively represent biological features for relevant entities in bioinformatics (Min et al., 2017; Zhang et al., 2018d; Peng et al., 2019; Zeng et al., 2019), such as information relevant to LPI prediction (Xiao et al., 2017; Shen et al., 2019; Zhu et al., 2019). More importantly, although deep learning demonstrated promising performance, it is not a silver bullet in LPI prediction. There still exist many challenges in LPI identification, such as the imbalanced nature of LPI data, limited LPI data, appropriate architecture selection, hyper parameter selection, and interpretation of learning results (Min et al., 2017). Therefore, solving these problems is the key to promoting deep learning-based LPI prediction models in future research.

Particularly, deep learning can be combined with PU learning and improve the performance of computational models (Bepler et al., 2018; Pati et al., 2018). For example, Bepler et al., 2018 designed the first particle-picking framework, Topaz. Topaz combined a convolutional neural network with a generalized-expectation-binomial-based objective function. The convolutional neural network was used to train classification models using only positive and unlabeled samples. Meanwhile, the generalized-expectation-binomial-based objective function was used to learn model parameters based on positive and unlabeled samples. Topaz utilized convolutional neural network classifiers to fit labeled particles (samples) and the remaining unlabeled samples based on the minibatched stochastic gradient decent method. Deep learning methods based on PU learning provide valuable insight and may be a starting point for deep learning applied to LPI prediction in future research.

### Capturing LPI Candidates for New LncRNAs/Proteins

Network-based methods can be applied to an LPI network that has least one link between two nodes. For a bipartite network especially, network-based methods require that each node in the network has at least two linkages. That is to say, network-based methods cannot discover possible proteins for any lncRNA–protein pair without any known reachable paths in the LPI network (Ge et al., 2016; Zhang et al., 2018c). These lncRNAs/proteins without any interaction information are regarded as new lncRNAs/proteins (Zhang et al,. 2018c).

Given a known LPI dataset, we aim to predict (S1) LPIs between known lncRNAs and known proteins; (S2) LPIs between new lncRNAs and known proteins; (S3) LPIs between known lncRNAs and new proteins; and (S4) LPIs between new lncRNAs and new proteins. S1 has the most abundant association information, S2 and S3 have less data, and S4 has the least data. Computational models appropriate for S2 can still be applied to S3, and vice versa.

To the best of our knowledge, SFPEL-LPI provided by Zhang et al. (2018c) may be one of the rare computational methods for predicting possible LPIs for new lncRNAs/proteins. Although few computational models can be applied to the last three situations, some methods have been designed to solve similar problems in other areas in bioinformatics, and thus provide some clues for LPI prediction. For example, Shi et al. (2015) enhanced the similarity measures and introduced the concept of a “super-target” to capture the missing interactions for new drugs/targets. Furthermore, Chen et al. (2016b) exploited a miRNA–disease association prediction model based on within and between scores (WBSMDA) to uncover possible miRNA–disease associations for new miRNAs/diseases. These solutions provide clues for capturing LPI candidates for new lncRNAs/proteins.

### Cross-Validation

Inspired by the evaluation methods proposed by Park and Marcotte (2012) and Chen et al. (2015), the test samples of LPIs could be categorized into four different groups: C1 is composed of the test samples sharing both lncRNAs and proteins with the training samples; C2 is composed of the test samples sharing only lncRNA with the training samples; C3 is composed of the test samples sharing only proteins with the training samples; and C4 is composed of the test samples sharing neither lncRNAs nor proteins with the training samples (Chen et al. (2015)). Therefore, it is vital to give cross-validation results under the above four independent test classes for LPI prediction.

## Author Contributions

LP and FL contributed equally to this work. LP, FL, XD, CP, and LZ introduced LPI data repositories and computational models. LP and FL wrote the paper. XL and YM revised original draft. LP, JY, GT, and LZ discussed the computational models and gave conclusion and further research. All authors read and approved the final manuscript.

## Funding

This research was funded by the Natural Science Foundation of China (Grant 61803151), the Natural Science Foundation of Hunan province (Grant 2018JJ2461, 2018JJ3570), and the Project of Scientific Research Fund of Hunan Provincial Education Department (Grant 17A052). 

## Conflict of Interest

Authors GT and JY were employed by the company Geneis (Beijing) Co. Ltd.

The remaining authors declare that the research was conducted in the absence of any commercial or financial relationships that could be construed as a potential conflict of interest.
